# The path to universal health coverage in five African and Asian countries: examining the association between insurance status and health-care use

**DOI:** 10.1016/S2214-109X(23)00510-7

**Published:** 2023-12-11

**Authors:** Emily Odipo, Prashant Jarhyan, Jacinta Nzinga, Dorairaj Prabhakaran, Amit Aryal, Emma Clarke-Deelder, Sailesh Mohan, Moshabela Mosa, Munir Kassa Eshetu, Todd P Lewis, Neena R Kapoor, Margaret E Kruk, Günther Fink, Emelda A Okiro

**Affiliations:** a Population and Health Impact Surveillance Group, Kenya Medical Research Institute–Wellcome Trust Research Programme, Nairobi, Kenya; b Health Economics Research Unit, Kenya Medical Research Institute–Wellcome Trust Research Programme, Nairobi, Kenya; c Public Health Foundation of India, Gurgaon, India; d Swiss Tropical and Public Health Institute, Basel, Switzerland; e University of Basel, Basel, Switzerland; f University of KwaZulu-Natal, Durban, South Africa; g Ministry of Health of Ethiopia, Addis Ababa, Ethiopia; h Department of Global Health and Population, Harvard T H Chan School of Public Health, Boston, MA, USA; i Centre for Tropical Medicine and Global Health, Nuffield Department of Medicine, University of Oxford, Oxford, UK

## Abstract

Despite major efforts to achieve universal health coverage (UHC), progress has lagged in many African and Asian countries. A key strategy pursued by many countries is the use of health insurance to increase access and affordability. However, evidence on insurance coverage and on the association between insurance and UHC is mixed. We analysed nationally representative cross-sectional data collected between 2022 and 2023 in Ethiopia, Kenya, South Africa, India, and Laos. We described public and private insurance coverage by sociodemographic factors and used logistic regression to examine the associations between insurance status and seven health-care use outcomes. Health insurance coverage ranged from 25% in India to 100% in Laos. The share of private insurance ranged from 1% in Ethiopia to 13% in South Africa. Relative to the population with private insurance, the uninsured population had reduced odds of health-care use (adjusted odds ratio 0·68, 95% CI 0·50–0·94), cardiovascular examinations (0·63, 0·47–0·85), eye and dental examinations (0·54, 0·42–0·70), and ability to get or afford care (0·64, 0·48–0·86); private insurance was not associated with unmet need, mental health care, and cancer screening. Relative to private insurance, public insurance was associated with reduced odds of health-care use (0·60, 0·43–0·82), mental health care (0·50, 0·31–0·80), cardiovascular examinations (0·62, 0·46–0·84), and eye and dental examinations (0·50, 0·38–0·65). Results were highly heterogeneous across countries. Public health insurance appears to be only weakly associated with access to health services in the countries studied. Further research is needed to improve understanding of these associations and to identify the most effective financing strategies to achieve UHC.

This is the third in a **Series** of six papers about the People's Voice Survey on Health System Performance. All papers in the Series are available at www.thelancet.com/series/peoples-voice-survey

## Introduction

Universal health coverage (UHC) is defined as the possibility for all individuals to access quality health care at an affordable cost.^1^ The global commitment towards UHC seems substantial at the moment. However, actual progress towards UHC has been slow in some regions, particularly in Africa and Asia, where the coverage of essential health services is low in many settings.^2^

One of the key tools that many countries apply to achieve UHC is health insurance. Although the specific design and implementation of health insurance schemes vary substantially between countries and contexts, all insurance programmes aim to increase timely access to health-care services^3^ and to minimise the financial burden on individuals and families.^2^, ^4^, ^5^, ^6^, ^7^ The definition of health insurance that we followed in this Series paper was (1) the formal legal definition of a health insurance programme in the country and (2) a programme that provided access to all facilities, independent of whether the facility was government-owned or privately owned. Current evidence suggests that well designed insurance programmes can reduce delays in seeking care, induce earlier detection and treatment of illnesses, and contribute to improved management of chronic conditions, user experiences, and most importantly, health outcomes.^8^, ^9^, ^10^, ^11^, ^12^, ^13^ Finally, insurance can contribute to the sustainability and equity of health systems by creating a pool of funds that can be used to finance health-care services for the entire population.^14^, ^15^

A 2012 review focusing specifically on Africa and Asia^16^ suggested that community-based health insurance and social health insurance have generally positive effects on service use and financial protection. According to the latest WHO estimates, 11 million people in Africa and 550 million people in Asia face catastrophic health expenditures annually.^17^, ^18^ The proportion of the population covered by health insurance varies substantially across Africa and Asia. In Africa, only 17% of the population has access to health insurance, with less than 1% of the populations covered in some countries, such as Niger.^19^ In Asia, the proportion of the population covered by health insurance ranges from 1% in Afghanistan to 98% in Japan.^20^

**Key messages**

• In efforts to achieve universal health coverage, many countries in Asia and Africa have scaled up health insurance programmes.
• Despite the substantial evidence regarding the potential benefits of health insurance in terms of improved access and affordability of health-care services, evidence of insurance effect on care use in Africa and Asia is scarce.
• Insurance coverage is highly heterogeneous within and across countries, and the general associations between enrolment in national health insurance programmes and service use indicators are weak.
• Private insurance enrolment is generally more strongly associated with health-care access than enrolment in public insurance schemes.
• Current public insurance programmes might be insufficient to ensure adequate access to high-quality health-care services.
• New and more comprehensive, locally customised approaches are likely to be needed to reach the ambitious universal health care targets in Asian and African countries.

Although many studies have analysed the effects of insurance on financial risk protection and service coverage in low-income settings,^16^, ^21^ evidence on the contributions of current insurance programmes towards the ambitious UHC goals is scarce and mixed. In practice, insurance alone might not be sufficient to achieve access to quality care, which depends on a large range of factors, such as health-care infrastructure, provider availability, and local culture.^3^, ^22^, ^23^

Recent evidence from sub-Saharan Africa suggests that countries that have implemented national health insurance have generally not performed better than countries without such programmes in terms of resource mobilisation, service coverage, or financial protection.^2^ Positive effects of national health insurance on service use have been documented for Rwanda,^24^ South Africa,^25^ and Ghana.^26^ Conversely, two papers from Kenya emphasise that Kenya's UHC index is low despite its National Health Insurance Fund.^10^, ^12^

This study aims to provide an overview of the current state of health insurance in some African and Asian countries, focusing on how coverage varies across and within countries, and the association between insurance status and use of key preventive health-care services and health system competence. Our main hypothesis was that having a health insurance card would increase access to care overall, and particularly access to quality care. The results can inform the ongoing global dialogue on how social determinants of health influence UHC progress and how different insurance schemes influence UHC outcomes.

## Study design

The People's Voice Survey (PVS) assesses health system quality, confidence, use, and other topics from the perspective of the general adult population (appendix 1). This study focuses on available PVS data in Africa and Asia: Ethiopia, Kenya, India, Laos, and South Africa.

All five surveys conducted were designed to be nationally representative in each country. For all PVS surveys, a minimum sample size of 1000 was targeted to provide prevalence estimates with a standard error of 1·5 percentage points. To be able to conduct stratified analysis with similar precision, a target sample size of 2000 was set for all five surveys analysed here. The full sample was obtained using mobile phones in countries with over 80% of households owning at least one mobile phone (ie, India, Laos, and South Africa). In Ethiopia and Kenya, the mobile phone sample was supplemented with a sample of face-to-face interviews focused on rural areas to increase the representativeness of the sample. Appendix 1 shows details on the survey modalities in each country. Random digit dialling and telephone interview were used to recruit and select participants in all countries apart from Ethiopia, where a known-list sampling approach was used. In Ethiopia and Kenya, primary sampling units were selected using multistage cluster sampling and then random walk was used to identify households for face-to-face interviews (appendix 1).

All data were collected between May 9, 2022, and April 3, 2023. Interviews lasted a mean of 23 min (SD 12), with 10 498 (94%) of 11 161 interviews performed via mobile phones and 663 (6%) of 11 161 interviews conducted face to face (in Kenya and Ethiopia). Verbal informed consent was obtained before the interview began. Ethical approval for all data collection was obtained from national ethical committees in each country.

The five countries in this study were selected because they are the first countries in Africa and Asia to complete the PVS. These countries represent various health insurance models summarised in table 1. Whereas a national health insurance scheme covers the entire population in Laos, national health insurance covers only a part of the population in Ethiopia, India, and Kenya; in all five countries, private insurance plans co-exist with these public insurance programmes. In South Africa, the public health system is universally free at the point-of-service for the entire population and is largely funded by tax revenue. A new national health insurance is currently being proposed in South Africa, with a new health insurance bill in advanced stages of approval by the South African Government. Private health insurance provides access to additional private facilities. Appendix 1 (p 4) provides further country characteristics at the time of data collection.

**Table 1:** Health insurance programmes by country

	**Model**	**Definition of variables**	**Benefits package**
**Ethiopia**			
Public	Primarily government-financed health-care system; public insurance model (ie, Ethiopian Health Insurance Agency) available for civil servants and some private employees	National or community-based health insurance	Community-based health insurance benefits package, including all family health services and curative care (ie, inpatient services, outpatient services, and acute illnesses)
Private	Little coverage	Employer-provided health insurance or private health insurance	Private insurance plans vary widely with respect to coverage and co-pays
**Kenya**			
Public	Primarily government-funded health-care system; public insurance model (ie, NHIF) for both formal sector and informal sector employees; an automatic income-rated monthly deduction for formal sector employees, whereas informal sector individuals pay premiums voluntarily	NHIF only	The national health scheme is the main scheme under the NHIF and includes inpatient, outpatient, maternity, renal dialysis, oncological, surgical, radiological, mental or behavioural, and emergency care and overseas treatment benefits packages; other schemes under NHIF include the enhanced scheme (ie, an enhanced form of national health scheme cover including a pension scheme for retired public officers, among others), Linda mama (ie, free maternity service programme administered through the NHIF), and Edu Afya (ie, a comprehensive medical scheme that covers all public secondary school students [aged approximately 15–18 years] with NHIF registration and who are recorded under the National Education Management Information System)
Private	A few private health facilities; private insurance model offered by commercial insurance companies, for which members pay premiums voluntarily	Private insurance only, both NHIF and private insurance, or company-provided insurance	Private insurance packages vary on the basis of agreement between the insurance provider and an individual or the company paying for the insurance and mainly cover inpatient and outpatient services to defined limits depending on premium payment
**South Africa**			
Public	Dual system with care freely available in the public sector; national health insurance scheme covering everybody to be implemented	Not yet implemented	To be defined
Private	Dual system with private insurance providing access to additional private facilities	Hospital plan or hospital plan with day-to-day benefits	Access to all public and private facilities; co-pays vary by insurance provider
**Laos**			
Public	Health care is primarily provided through government facilities; the NHI scheme initiated in 2016 provides social health protection to the population with nearly universal coverage	NHI	Access to all public facilities with small co-payments; free services for pregnant women, children, and low-income households
Private	Additional private insurance is available	NHI plus additional private insurance	Access to private and public facilities; co-pays vary by plan
**India**			
Public	For people living below the poverty line, central and state government employees, and employees of large firms	Central Government Health Scheme, State Government Health Scheme, Employee State Health Insurance Scheme, and Ayushman Bharat Pradhan Mantri Jan Arogya Yojana	The Central Government Health Scheme provides free comprehensive medical care to 4·2 million central government employees, pensioners, and their dependent family members through its polyclinics and hospitals and empanelled private hospitals; the State Government Health Scheme provides free comprehensive medical care to state government employees, pensioners, and dependents in state government clinics and hospitals and empanelled private hospitals; the Employee State Health Insurance Scheme is a social insurance scheme providing free comprehensive medical care to 120 million beneficiaries (ie, employees and their families of any establishment with ten or more people employed and drawing wages up to ₹21 000 per month as defined in the Employee State Health Insurance Scheme act); the Ayushman Bharat Pradhan Mantri Jan Arogya Yojana provides coverage of ₹500 000 per family per year to more than 500 million vulnerable people and people with a low income for inpatient secondary and tertiary care
Private	Offered by commercial insurance companies for individuals and groups	Employer-provided health insurance or private health insurance	Employer-provided (for those employees not covered under the Employee State Health Insurance Scheme act) or privately purchased; access to empanelled private care mainly for inpatient or emergency care with cashless or co-payment according to plan

NHI=National Health Insurance. NHIF=National Health Insurance Fund.

## Variables

We considered six indicators of health-care service use and system competence: general use of health services, defined as reporting at least one visit to a health-care facility in the past 12 months; a binary indicator for unmet need for care; use of mental health care in the past 12 months; having received cervical cancer screening or mammography in the past 12 months; having received cardiovascular examinations, including blood pressure, glucose, and cholesterol tests, in the past 12 months; and having received an eye or dental examination in the past 12 months. Unmet need was defined as a respondent indicating a health problem in the past 12 months that needed medical attention who did not receive care from a provider. Cervical cancer screening questions were asked only to women older than 18 years and mammogram questions were asked only to women older than 50 years. Further details on the survey questions on use and health system competence are shown in appendix 2 (p 2).

We also analysed confidence in the ability to afford care as a separate outcome (1 if confident of receiving and affording care if needed and 0 otherwise). These indicators were selected because they are sensitive to insurance and have been used in previous papers to explain the role of insurance in motivating needed health-care use. The concept underlying these indicators is described in The *Lancet Global Health* Commission on High Quality Health Systems as detailed in appendix 1 (p 3).^23^

Health insurance status was the primary independent variable. We divided the study population into three groups: uninsured individuals (ie, people with no health insurance card), individuals with public health insurance, and individuals with private health insurance or other (ie, voluntary or employer-based) health insurance.

We classified other independent variables into three types, namely predisposing, enabling, and need variables, on the basis of Andersen and Newman's health-care use model,^27^ which has directed studies on the factors linked to health-care use.^27^, ^28^ In this framework, predisposing factors are characteristics that make individuals more likely to use a service ex-ante (ie, before onset of an illness). In the context of health insurance, the most obvious predisposing factors are age, gender, and highest education level. Factors that allow an individual to fulfil their health-care needs when they are ill are referred to as enabling factors in this framework.^27^ Enabling factors in this context are those related to access and affordability, which we approximate with urban residence and household income. Variables predicting need included subjective need (ie, self-perceived need, including general health and mental health) and objective need (ie, evaluated by health professionals), such as chronic health status. We further describe our conceptual framework and variables in appendix 2 (p 3).

## Data analysis

Across all the countries, health insurance was categorised as public, private, and uninsured. In Laos, public health insurance is provided to everybody, whereas in South Africa, public health insurance is not currently available. In both of these countries, we can thus only compare two groups.

First, we calculated the proportions of the population in each country with no insurance, private insurance, and public insurance. We assessed associations between health insurance coverage and demographic characteristics, including age, gender, urban residence, highest education level, and household income, using Pearson's χ^2^ tests. Additionally, we calculated proportions of health insurance against type of health-care facility and main reason for choice of health-care facility as a usual source of care.

Second, we conducted a descriptive analysis of the seven outcomes: general use of health services, unmet need for care, mental health care, cancer screening, cardiovascular examinations, eye and dental examinations, and confidence in ability to receive and afford care. We described use patterns in each country and then assessed bivariate associations between use and predisposing factors, enabling factors, and need variables as described.

Last, we used logistic regression models to explore the general association between insurance and our outcome variables, both unconditional and conditional on other covariates. Separate models were constructed for each outcome variable: health-care visits, unmet need, mental health care, cancer screening, general examinations, eye and dental examinations, and confidence in the ability to afford care. Adjusted models included controls for age, gender, general health, mental health, chronic health, urban residence, education level, and household income. Models were first estimated for each country separately and then in the pooled sample. Given the observational nature of the data, these models were intended to be hypothesis-generating and not to generate causal evidence on insurance effect. Forest plots were used to summarise the pooled models. An α level of 0·05 was applied for all analyses, and all results were weighted using sampling weights. Analysis was done using the R statistical software package, version 4.2.1.

## Insurance coverage status

**Figure 1:**
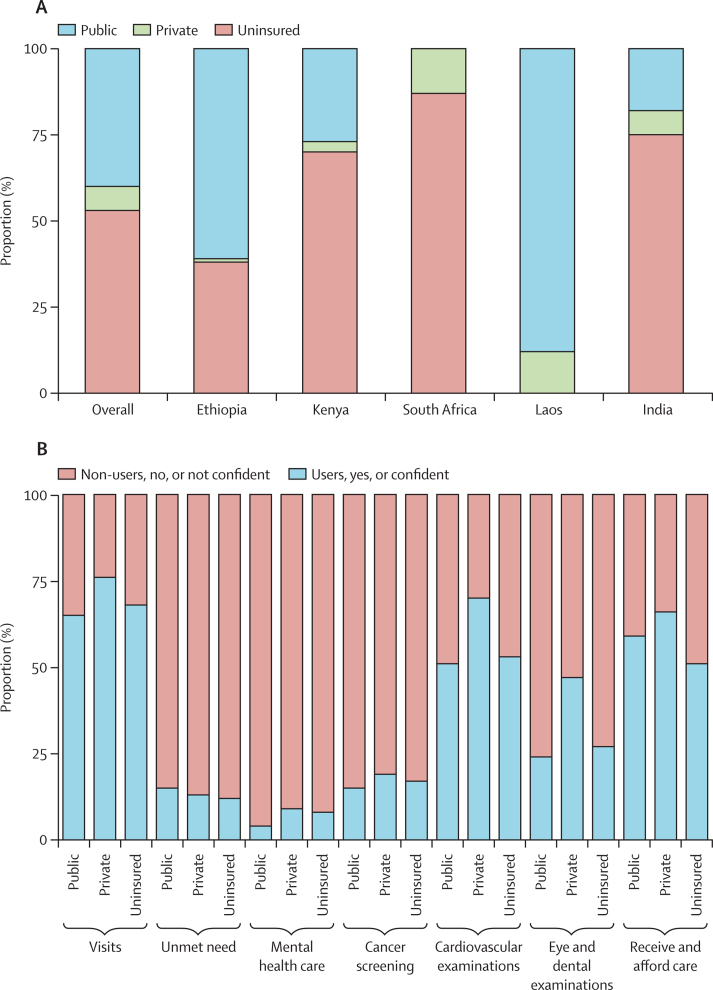
Types of insurance by country and health-care use status (A) Type of insurance by country. Insurance status is categorised as public, private (including private insurance and other voluntary or employer-offered schemes), and uninsured. Laos is assumed to have 100% public health insurance, whereas South Africa does not have public insurance. (B) Type of insurance by health-care use status. Insurance status was categorised as public, private (including private insurance and other voluntary or employer-offered schemes), and uninsured. Visits were categorised as users and non-users. Unmet health-care need, mental health care, cancer screening, cardiovascular examination, and eye and dental examination were categorised as a yes if participants received the service and a no if participants did not receive the service. The ability to receive and afford care was categorised as confident or not confident.

Our analytic sample included 11 131 respondents across the five countries. Whereas the entire population was covered by health insurance in Laos, public health insurance was not available in South Africa. Public health insurance coverage was 61% in Ethiopia and below 50% in Kenya and India (figure 1A). Public health insurance was the most common type of health insurance among insured respondents in most countries, with the exception of South Africa, which does not have public health insurance. The two countries with the highest private health insurance coverage in our sample were Laos (232 [12%] of 1988 people covered) and South Africa (272 [13%] of 2028 people covered).

Table 2, Table 3 show the insurance coverage status by sociodemographic, economic, and health characteristics. In the pooled estimates, public insurance coverage was lowest among individuals aged 18–29 years and 30–39 years and highest among individuals aged 50–59 years, and private insurance coverage was lowest among individuals aged 18–29 years and highest among individuals aged 60 years or older. In all countries, private insurance coverage was highest for people in the most educated group. In Laos (with universal public health insurance) and South Africa (with a universally free public health system), additional private health insurance was most common among individuals aged 60 years or older, residents from urban areas, and people in the highest income group. More than half of the population across all age groups are uninsured in India, South Africa, and Kenya, with increasing insurance coverage among older adults.

**Table 2:** Private and public insurance enrolment by socioeconomic characteristics

		**Ethiopia**				**Kenya**				**South Africa**			
		Uninsured (n=975)	Public (n=1536)	Private (n=37)	p value	Uninsured (n=1361)	Public (n=589)	Private (n=70)	p value	Uninsured (n=1705)	Public (n=0)	Private (n=253)	p value
Age group, years		..	..	..	0·30	..	..	..	<0·0001	..	..	..	<0·0001
	18–29	438/1102 (40%)	652/1102 (59%)	12/1102 (1%)	..	642/821 (78%)	167/821 (20%)	12/821 (1%)	..	503/562 (90%)	0/562	59/562 (10%)	..
	30–39	276/625 (44%)	336/625 (54%)	13/625 (2%)	..	310/505 (61%)	167/505 (33%)	28/505 (6%)	..	471/521 (90%)	0/521	50/521 (10%)	..
	40–49	129/395 (33%)	258/395 (65%)	8/395 (2%)	..	218/359 (61%)	128/359 (36%)	13/359 (4%)	..	348/393 (89%)	0/393	45/393 (11%)	..
	50–59	51/188 (27%)	135/188 (72%)	2/188 (1%)	..	114/193 (59%)	67/193 (35%)	12/193 (6%)	..	179/208 (86%)	0/208	29/208 (14%)	..
	≥60	82/238 (34%)	155/238 (65%)	1/238 (<1%)	..	77/141 (55%)	60/141 (43%)	4/141 (3%)	..	204/274 (74%)	0/274	70/274 (26%)	..
Gender		..	..	..	0·60	..	..	..	<0·0001	..	..	..	0·80
	Male	492/1303 (38%)	786/1303 (60%)	25/1303 (2%)	..	627/1016 (62%)	352/1016 (35%)	37/1016 (4%)	..	826/946 (87%)	0/946	120/946 (13%)	..
	Female	483/1245 (39%)	750/1245 (60%)	12/1245 (1%)	..	735/1005 (73%)	237/1005 (24%)	33/1005 (3%)	..	879/1012 (87%)	0/1012	133/1012 (13%)	..
Area of residence		..	..	..	<0·0001	..	..	..	<0·0001	..	..	..	<0·0001
	Rural	543/1793 (30%)	1248/1793 (70%)	2/1793 (<1%)	..	1016/1316 (77%)	271/1316 (21%)	29/1316 (2%)	..	571/611 (93%)	0/611	40/611 (7%)	..
	Urban	432/755 (57%)	288/755 (38%)	35/755 (5%)	..	345/705 (49%)	319/705 (45%)	41/705 (6%)	..	1134/1348 (84%)	0/1348	214/1348 (16%)	..
Income group		..	..	..	<0·0001	..	..	..	<0·0001	..	..	..	<0·0001
	Low or middle	551/1747 (32%)	1177/1747 (67%)	19/1747 (1%)	..	1077/1417 (76%)	318/1417 (22%)	22/1417 (2%)	..	1377/1453 (95%)	0/1453	76/1453 (5%)	..
	Highest	424/802 (53%)	359/802 (45%)	19/802 (2%)	..	285/603 (47%)	271/603 (45%)	47/603 (8%)	..	328/505 (65%)	0/505	177/505 (35%)	..
Education		..	..	..	<0·0001	..	..	..	<0·0001	..	..	..	<0·0001
	None or primary	660/2022 (33%)	1341/2022 (66%)	21/2022 (1%)	..	965/1273 (76%)	288/1273 (23%)	20/1273 (2%)	..	1027/1091 (94%)	0/1091	64/1091 (6%)	..
	Secondary	160/312 (51%)	148/312 (47%)	4/312 (1%)	..	328/522 (63%)	183/522 (35%)	11/522 (2%)	..	534/645 (83%)	0/645	111/645 (17%)	..
	Post-secondary	155/215 (72%)	48/215 (22%)	12/215 (6%)	..	69/226 (31%)	118/226 (52%)	39/226 (17%)	..	144/222 (65%)	0/222	78/222 (35%)	..

Private insurance includes private and other voluntary or employer-offered schemes. All counts were weighted.

**Table 3:** Private and public insurance enrolment by socioeconomic characteristics

		**Laos**				**India**				**Pooled**			
		Uninsured (n=0)	Public (n=1548)	Private (n=220)	p value	Uninsured (n=1153)	Public (n=237)	Private (n=110)	p value	Uninsured (n=3490)	Public (n=5615)	Private (n=690)	p value
Age group, years		..	..	..	0·40	..	..	..	0·0079	..	..	..	0·0073
	18–29	0/548	488/548 (89%)	60/548 (11%)	..	467/545 (86%)	50/545 (9%)	28/545 (5%)	..	2050/3578 (57%)	1356/3578 (38%)	172/3578 (5%)	..
	30–39	0/359	313/359 (87%)	46/359 (13%)	..	217/306 (71%)	70/306 (23%)	19/306 (6%)	..	1275/2317 (55%)	885/2317 (38%)	157/2317 (7%)	..
	40–49	0/400	345/400 (86%)	55/400 (14%)	..	239/322 (74%)	57/322 (18%)	26/322 (8%)	..	934/1870 (50%)	789/1870 (42%)	147/1870 (8%)	..
	50–59	0/298	269/298 (90%)	29/298 (10%)	..	111/155 (72%)	16/155 (10%)	28/155 (18%)	..	455/1043 (44%)	487/1043 (47%)	101/1043 (10%)	..
	≥60	0/163	133/163 (82%)	30/163 (18%)	..	118/170 (69%)	44/170 (26%)	8/170 (5%)	..	481/987 (49%)	393/987 (40%)	113/987 (11%)	..
Gender		..	..	..	0·80	..	..	..	0·80	..	..	..	0·40
	Male	0/877	766/877 (87%)	111/877 (13%)	..	630/832 (76%)	140/832 (17%)	62/832 (7%)	..	2575/4976 (52%)	2045/4976 (41%)	356/4976 (7%)	..
	Female	0/890	781/890 (88%)	109/890 (12%)	..	523/669 (78%)	98/669 (15%)	48/669 (7%)	..	2620/4820 (54%)	1866/4820 (39%)	334/4820 (7%)	..
Area of residence		..	..	..	<0·0001	..	..	..	0·0059	..	..	..	<0·0001
	Rural	0/1187	1082/1187 (91%)	105/1187 (9%)	..	569/754 (75%)	148/754 (20%)	37/754 (5%)	..	2700/5662 (48%)	2749/5662 (49%)	213/5662 (4%)	..
	Urban	0/582	466/582 (80%)	116/582 (20%)	..	584/746 (78%)	89/746 (12%)	73/746 (10%)	..	2495/4134 (60%)	1161/4134 (28%)	478/4134 (12%)	..
Income group		..	..	..	0·0047	..	..	..	0·40	..	..	..	<0·0001
	Low or middle	0/892	812/892 (91%)	80/892 (9%)	..	995/1300 (77%)	215/1300 (17%)	90/1300 (7%)	..	4000/6809 (59%)	2523/6809 (37%)	286/6809 (4%)	..
	Highest	0/877	736/877 (84%)	141/877 (16%)	..	158/200 (79%)	22/200 (11%)	20/200 (10%)	..	1195/2986 (40%)	1387/2986 (46%)	404/2986 (14%)	..
Education		..	..	..	<0·0001	..	..	..	0·10	..	..	..	<0·0001
	None or primary	0/940	863/940 (92%)	77/940 (8%)	..	382/501 (76%)	95/501 (19%)	24/501 (5%)	..	3034/5828 (52%)	2587/5828 (44%)	207/5828 (4%)	..
	Secondary	0/548	476/548 (87%)	72/548 (13%)	..	328/417 (79%)	67/417 (16%)	22/417 (5%)	..	1350/2444 (55%)	874/2444 (36%)	220/2444 (9%)	..
	Post-secondary	0/278	208/278 (75%)	70/278 (25%)	..	442/580 (76%)	75/580 (13%)	63/580 (11%)	..	810/1522 (53%)	449/1522 (30%)	263/1522 (17%)	..

Private insurance includes private and other voluntary or employer-offered schemes. All counts were weighted.

Health-care use among people with various sociodemographic, economic, and health characteristics and insurance statuses are shown in appendix 2 (p 5). Overall, 6542 (67%) of 9733 participants reported visiting the health system for health care in the past 12 months. 5201 (53%) participants received cardiovascular examinations. Compared with cardiovascular examinations, use rates were lower for eye and dental examinations (2629 [27%] of 9721 respondents), cancer screening (782 [16%] of 4756 respondents), and mental health care (638 [7%] of 9660 respondents). 5356 (55%) of the 9690 respondents were confident that they could receive and afford care if needed, and few (1319 [14%] of 9763 respondents) reported unmet health-care needs. Generally, use was highest among respondents aged 60 years or older, except for health-care visits, where use was highest among people aged 50–59 years. Female respondents also reported more health-service use than male respondents, except for eye and dental examinations. Use was also higher among residents of urban areas than among residents of rural areas.

Relative to people who were uninsured, individuals with public insurance were more likely to report unmet need and confidence in ability to get and afford care. Individuals with private health insurance reported highest usage of health services (524 [76%] of 685 respondents with private insurance *vs* 2520 [65%] of 3889 respondents with public insurance *vs* 3498 [68%] of 5159 respondents who were uninsured). These differences appear to be primarily driven by general cardiovascular-related examinations (484 [70%] of 689 respondents *vs* 1991 [51%] of 3900 respondents *vs* 2726 [53%] of 5184 respondents) and eye and dental examinations (321 [47%] of 689 respondents *vs* 935 [24%] of 3882 respondents *vs* 1373 [27%] of 5150 respondents). People with private insurance also reported higher ability to receive and afford care (444 [66%] of 677 respondents) than people with public health insurance (2283 [59%] of 3883 respondents) and people who were uninsured (2629 [51%] of 5130 respondents; figure 1B).

## Associations between health insurance type and health-care use

**Figure 2:**
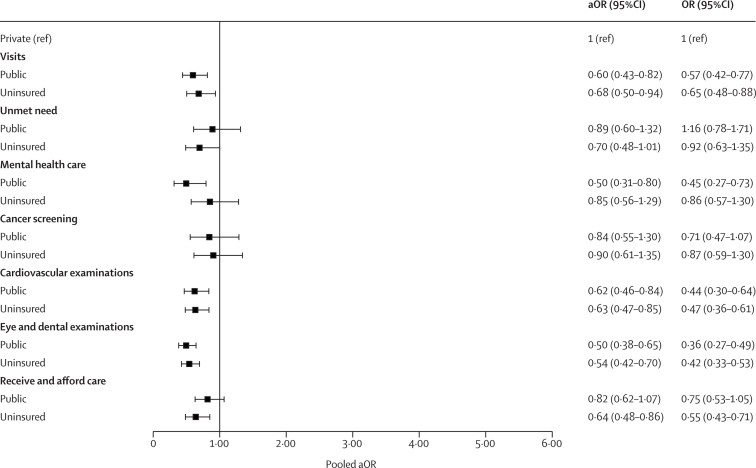
Adjusted associations between health insurance type and health-care use The health-care use outcomes considered were visits in the past 12 months, unmet need, mental health care, cancer screening, cardiovascular examination, eye and dental examination, and ability to receive and afford care. aOR=adjusted odds ratio. OR=crude odds ratio.

In the fully adjusted models, respondents with public insurance (adjusted odds ratio 0·60, 95% CI 0·43–0·82) and respondents who were uninsured (0·68, 0·50–0·94) were less likely to use health services than people with private health insurance (figure 2). Participants with public insurance were also less likely to receive mental health care (0·50, 0·31–0·80), a cardiovascular examinations (0·62, 0·46–0·84), and eye and dental examinations (0·50, 0·38–0·65) than people with private health insurance. Uninsured individuals were less likely to receive cardiovascular examinations (0·63, 0·47–0·85) than people with private insurance. Although not significant, health-care seeking for cancer screening in women was higher for people with private health insurance than people without insurance (0·90, 0·61–1·35) and public health insurance (0·84, 0·55–1·30). Individuals with private health insurance had higher confidence in their ability to receive and afford health care than people without insurance (0·64, 0·48–0·86; figure 2).

Use patterns varied substantially across countries (table 4). In Ethiopia, the differences between uninsured individuals and people with public insurance were generally small. Individuals with private insurance had slightly higher use rates, but most differences (except for eye and dental examinations for people with public insurance) were not significant in adjusted models. In India and Kenya, uninsured populations generally used health services at substantially lower rates than individuals with private health insurance. Relative to private health insurance, public health insurance was generally associated with reduced access to care in Kenya. In Laos, individuals with private health insurance generally appeared to use health services more frequently than those covered by public health insurance. In South Africa, individuals with private health insurance generally appeared to use health services more frequently than those who were uninsured (except for cancer screening). The differences in use of eye and dental examinations, and also in confidence in the ability to receive and afford care, were significant in both Laos and South Africa.

**Table 4:** Country-specific associations between health insurance type and health-care use

	**Ethiopia**		**Kenya**		**South Africa**		**Laos**		**India**	
	OR (95% CI)	aOR (95% CI)	OR (95% CI)	aOR (95% CI)	OR (95% CI)	aOR (95% CI)	OR (95% CI)	aOR (95% CI)	OR (95% CI)	aOR (95% CI)
**Visits**										
Private+	1 (ref)	1 (ref)	1 (ref)	1 (ref)	1 (ref)	1 (ref)	1 (ref)	1 (ref)	1 (ref)	1 (ref)
Public	0·53 (0·15–1·81)	0·64 (0·22–1·84)	0·38 (0·17–0·84)	0·38 (0·17–0·85)	NA	NA	0·65 (0·38–1·11)	0·74 (0·43–1·29)	1·34 (0·61–2·93)	1·68 (0·77–3·67)
Uninsured	0·53 (0·16–1·81)	0·52 (0·19–1·45)	0·58 (0·27–1·26)	0·41 (0·19–0·91)	0·71 (0·45–1·13)	0·69 (0·41–1·16)	NA	NA	0·51 (0·27–0·98)	0·55 (0·30–1·01)
**Reported unmet need**										
Private+	1 (ref)	1 (ref)	1 (ref)	1 (ref)	1 (ref)	1 (ref)	1 (ref)	1 (ref)	1 (ref)	1 (ref)
Public	1·02 (0·34–3·03)	0·59 (0·18–1·97)	0·97 (0·45–2·09)	0·69 (0·31–1·55)	NA	NA	0·62 (0·37–1·03)	0·63 (0·37–1·09)	4·68 (1·31–16·8)	4·36 (1·24–15·3)
Uninsured	0·89 (0·35–2·28)	0·58 (0·18–1·85)	1·10 (0·54–2·26)	0·51 (0·25–1·05)	1·61 (0·70–3·69)	1·30 (0·60–2·80)	NA	NA	2·33 (0·85–6·39)	2·29 (0·77–6·81)
**Mental health care**										
Private+	1 (ref)	1 (ref)	1 (ref)	1 (ref)	1 (ref)	1 (ref)	1 (ref)	1 (ref)	1 (ref)	1 (ref)
Public	0·88 (0·27–2·89)	0·92 (0·27–3·09)	1·02 (0·42–2·51)	0·92 (0·38–2·26)	NA	NA	0·47 (0·14–1·60)	0·65 (0·17–2·48)	2·53 (0·94–6·81)	2·80 (0·97–8·07)
Uninsured	1·02 (0·30–3·48)	1·11 (0·33–3·71)	0·94 (0·37–2·40)	0·79 (0·31–2·06)	0·67 (0·39–1·15)	0·60 (0·32–1·13)	NA	NA	0·88 (0·39–2·00)	0·82 (0·31–2·14)
**Cancer screening**										
Private+	1 (ref)	1 (ref)	1 (ref)	1 (ref)	1 (ref)	1 (ref)	1 (ref)	1 (ref)	1 (ref)	1 (ref)
Public	1·34 (0·39–4·61)	3·08 (0·77–12·3)	0·56 (0·19–1·61)	0·50 (0·15–1·62)	NA	NA	0·76 (0·41–1·40)	0·56 (0·31–1·01)	4·49 (0·91–22·1)	2·00 (0·41–9·73)
Uninsured	2·29 (0·67–7·82)	4·04 (1·09–14·9)	0·27 (0·09–0·81)	0·19 (0·05–0·69)	1·51 (0·83–2·76)	1·17 (0·59–2·32)	NA	NA	1·33 (0·34–5·25)	0·49 (0·11–2·19)
**Cardiovascular examinations**										
Private+	1 (ref)	1 (ref)	1 (ref)	1 (ref)	1 (ref)	1 (ref)	1 (ref)	1 (ref)	1 (ref)	1 (ref)
Public	0·31 (0·11–0·85)	0·70 (0·30–1·65)	0·44 (0·21–0·93)	0·57 (0·28–1·15)	NA	NA	0·67 (0·40–1·12)	0·83 (0·49–1·40)	1·56 (0·73–3·35)	1·78 (0·87–3·66)
Uninsured	0·45 (0·18–1·11)	0·71 (0·32–1·56)	0·26 (0·13–0·53)	0·30 (0·15–0·61)	0·69 (0·46–1·04)	0·80 (0·52–1·21)	NA	NA	0·48 (0·25–0·92)	0·54 (0·30–0·99)
**Eye and dental examinations**										
Private+	1 (ref)	1 (ref)	1 (ref)	1 (ref)	1 (ref)	1 (ref)	1 (ref)	1 (ref)	1 (ref)	1 (ref)
Public	0·35 (0·16–0·75)	0·41 (0·19–0·87)	0·60 (0·32–1·15)	0·57 (0·29–1·11)	NA	NA	0·50 (0·33–0·75)	0·65 (0·43–0·98)	1·58 (0·71–3·54)	2·17 (0·82–5·74)
Uninsured	0·59 (0·28–1·25)	0·66 (0·33–1·32)	0·26 (0·14–0·48)	0·28 (0·14–0·55)	0·34 (0·24–0·50)	0·41 (0·27–0·62)	NA	NA	0·67 (0·34–1·31)	0·84 (0·35–2·05)
**Confidence in ability to receive and afford care**										
Private+	1 (ref)	1 (ref)	1 (ref)	1 (ref)	1 (ref)	1 (ref)	1 (ref)	1 (ref)	1 (ref)	1 (ref)
Public	1·67 (0·72–3·87)	1·49 (0·70–3·17)	0·77 (0·44–1·37)	0·82 (0·45–1·49)	NA	NA	0·43 (0·22–0·83)	0·50 (0·26–0·96)	1·85 (0·79–4·36)	1·86 (0·84–4·15)
Uninsured	1·77 (0·87–3·64)	1·66 (0·81–3·40)	0·59 (0·34–1·04)	0·66 (0·36–1·22)	0·63 (0·44–0·90)	0·52 (0·35–0·77)	NA	NA	1·44 (0·70–2·92)	1·39 (0·72–2·66)

Private insurance includes private and other voluntary or employer-offered schemes. aOR=adjusted odds ratio. NA=not applicable. OR=odds ratio.

Detailed regression results are provided in appendix 2 (pp 8–14). Generally, respondents with private health insurance were twice as likely to use private facilities compared with publicly insured or uninsured respondents. These differences in private versus public sector facility use were smallest in Laos and largest in South Africa (appendix 2 p 6). Public health facilities were largely preferred because of convenience in Kenya (590 [48%] of 1229 respondents who preferred public health facilities), South Africa (410 [41%] of 1003 respondents), and Laos (835 [59%] of 1405 respondents), whereas cost was the main reason in Ethiopia (595 [36%] of 1653 respondents) and India (106 [23%] of 454 respondents). Private health facilities were mostly preferred due to technical quality in all countries apart from Laos, where the main reason was interpersonal quality (appendix 2 p 7).

## Discussion

Establishing national health insurance schemes has become a primary strategy for many low-income and middle-income countries (LMICs) to achieve ambitious UHC goals.^2^ In this study, we used newly collected nationally representative data from five highly diverse LMICs to assess the population coverage of health insurance schemes and the extent to which health insurance schemes have helped to increase use of health care. To identify other factors predicting access to care, we relied on Andersen and Newman's health-care use model,^27^, ^28^ and tried to separate factors related to service access, individual predictors of service use, and health needs.

The analysis presented herein yields several key findings. First, consistent with previous literature,^29^ we identified that health insurance coverage is highly heterogeneous across LMICs, with overall insurance coverage ranging between 25% in India and full population coverage in Laos. In countries that have only partial insurance coverage, large variations were also observed in who was covered by insurance. Whereas insurance in Ethiopia often covered rural and vulnerable populations, the same is not true for Kenya, where absence of insurance is most common among populations living in rural locations and with low socioeconomic status. India has the lowest insurance coverage of the countries analysed in this study but has achieved this coverage relatively uniformly across all population groups, and insurance rates nowadays seem to be substantially higher than rates a few years ago.^30^ In general, gender differences were small; the only exception was in Kenya, where women were significantly more likely to be uninsured than men.

Second, in four of five countries analysed, public health insurance provided most insurance coverage among people with insurance. Private health insurance coverage was below 15% in all countries. Households that were enrolled in these private insurance programmes appeared to predominantly be located in urban areas, be classified in the highest income group, and have post-secondary education, as has been shown in other studies.^31^

Third, most adults in all countries analysed tended to use health services on a regular basis. 6542 (67%) of 9733 individuals indicated having used health services in the past year; however, unmet need clearly still exists, with one in seven respondents indicating that they were not able to receive care when needed in the year preceding the survey, and less than half of respondents indicating that they were not confident they could get and afford care when needed. Insufficient access to essential health services therefore remains a significant challenge, particularly for low-income households and individuals who are employed in the informal sector.

Fourth, public health insurance appears to be weakly associated with the use of health services. When we adjust for individual health and socioeconomic factors, there are only small differences between uninsured individuals and individuals with public insurance in terms of health service access; the only areas where individuals with public insurance appear to use services more are cardiovascular examinations and cancer screening, where usage rates are higher among publicly insured respondents in Kenya and Ethiopia than among the uninsured population. Remarkably, individuals with public insurance also do not appear more confident in getting and affording care than privately insured populations. Previous studies have reported a greater prevalence of unmet primary health-care needs among individuals without health insurance coverage.^32^ Financial barriers to accessing health-care services can lead to delayed or foregone care, increased morbidity and mortality rates, and exacerbation of health conditions.^3^

Public health insurance schemes are essential in ensuring that health-care services are accessible and affordable for vulnerable populations.^33^ For example, in some countries, such as Ghana and Rwanda, introducing public health insurance schemes has increased access to health-care services and reduced financial barriers.^24^, ^26^ However, the effectiveness of public health insurance schemes depends on various factors, such as funding, the service package offered, governance, and management. Effectiveness also requires sustained political commitment and investment.^33^

The usefulness of health insurance also depends on the health facilities most frequently used by patients. Most respondents in this study reported that they relied on public health facilities due to their convenience and low cost; private health facilities were largely chosen due to higher perceived service readiness and quality of care. Similar studies that were done in Ghana and Nigeria showed that private health facilities are preferred due to the quality of service and gratification received.^34^, ^35^ The results of this survey are crucial for policy makers because the survey focused on consumers of the health services, the importance of assessing health facilities, and restructuring for better health-care delivery in public health facilities. Additionally, in this study, respondents without insurance were less likely to report confidence in their ability to receive and afford care than those with private insurance. This result highlights that more emphasis should be put on service readiness and availability in health systems assessments.

In terms of use of the various health services, individuals with private health insurance appear to have generally higher usage of and confidence in being able to afford services than uninsured individuals as well as individuals with public health insurance in our sample. Private insurance appears less beneficial than public insurance in India, where the private insurance market is relatively underdeveloped.^36^ In Kenya and South Africa, private facilities, which might be unaffordable for the uninsured population, appear to provide better access to care than public facilities. Obtaining private insurance might generally reflect a higher willingness to pay for health services or higher health needs, so these differences should be interpreted carefully. Nevertheless, private insurance is achieving what most public insurance schemes are trying to achieve (ie, improved access to services).

A study from Togo suggests that mandatory health insurance can increase the likelihood of health-service use and advance countries closer to the goal of universal health care, and also emphasises the need to waive fees for lower-income earners.^37^ These suggestions are consistent with a systematic review by Shami and colleagues, which suggested that being insured increased the use of both inpatient and outpatient health services.^38^ A systematic review of studies focusing on low-income and middle-income countries also reported that health insurance cover improved health status and financial protection and increased health-care access, although findings were heterogeneous and not always consistent.^13^ Despite the increased uptake of public health insurance in sub-Saharan countries, experiences from Gabon, Ghana, and Rwanda clearly show that improving the effectiveness of existing health systems is crucial for achieving UHC targets.^2^

The generally weak link between public insurance and service use reported in this study is troubling and raises several questions. It seems possible that, in some settings, health insurance schemes are still relatively new and have not changed care-seeking behaviours. The finding might also be linked to ineffective public health insurance, as has been reported in similar contexts.^31^ Additionally, the general price differences created by public health insurance programmes are possibly not large enough to create systematic differences in care-seeking behaviour or people's perceptions of public facilities as poor.

The analysis presented herein has several limitations. First, and most obviously, we analysed data from only five countries, which are unlikely to fully represent the much larger group of LMICs. Second, all of the analyses presented here are cross-sectional in nature. Even though we controlled for a large set of covariates in our adjusted models, we cannot rule out residual confounding through other unobserved factors, which means that the associations presented here should thus not be interpreted causally. Third, our ability to compare uninsured with insured populations was limited by the fact that South Africa and Laos have public systems that de-facto cover everyone; thus, our insured versus uninsured comparisons are based on the other three countries alone. Fourth, relying on mobile phone surveys in some countries might imply that not all populations are fully covered in the data analysed. Fifth, we focused our analysis at a national level and, therefore, results might vary if done at subnational levels. Finally, our analysis focuses on only a subset of health services, particularly preventive health examinations. Results might differ if we considered other use outcomes, such as access to medicines or inpatient services.

Despite these limitations, we believe that the results presented here have important policy implications. In Kenya, for example, these findings can inform health system redesign and targeted interventions focused on bridging the gap in health insurance coverage between different groups, such as rural and urban populations. Our data suggest that the recent commitment towards national health insurance in South Africa could improve equity in terms of quality care access. India and Kenya might need additional and inclusive approaches for people who are uninsured to increase their health-care use for improved health outcomes. Our results clearly show that health insurance schemes do not always reach the populations that they are trying to support, and even if they do, these schemes might not necessarily help the targeted populations to get more access to health services. These challenges should be considered in ongoing efforts to achieve UHC in countries across Africa and Asia.

## Data sharing

Individual-level, de-identified data from the People's Voice Survey will be publicly available in mid-2024. Data will be available on the Harvard Dataverse. The survey instrument and data dictionary will be available on publication.

## Declaration of interests

We declare no competing interests.
