# Supplementary material for: The path to universal health coverage in five African and Asian countries: examining the association between insurance status and health-care use
Source: Lancet Glob Health. 2023 Dec 11;12(1):e123–33. doi: 10.1016/S2214-109X(23)00510-7 (PMC10716621; doi:10.1016/S2214-109X(23)00510-7)

THE LANCET
Global Health

Supplementary appendix 2

This appendix formed part of the original submission and has been peer reviewed. We post it as supplied by the authors.

Supplement to: Odipo E, Jarhyan P, Nzinga J, et al. The path to universal health coverage in five African and Asian countries: examining the association between insurance status and health-care use. *Lancet Glob Health* 2023; published online Dec 11. https://doi.org/10.1016/S2214-109X(23)00510-7.

Supplementary Appendix 2 to: *Path to Universal Health Coverage in Africa and Asia: Examining the Relationship Between Insurance Status and Healthcare Utilization*

Contents

Appendix 2: Supplementary Methodology and Results 2

Appendix 2 table 1: Survey questions on healthcare utilization outcomes and insurance 2

Appendix 2 figure 1: Anderson health utilization conceptual framework 3

Appendix 2 table 2: Descriptive characteristics by country 4

Appendix 2 table 3: Healthcare utilization by health insurance type and socio-demographic characteristics 5

Appendix 2 figure 2: Source of care facility type by insurance coverage 6

Appendix 2 figure 3: Main reason for choice of usual source of care per country 7

Appendix 2 figure 4: Estimated associations between type of health insurance and health care visits by countries: Odds ratio (OR) and adjusted odds ratio (aOR) 8

Appendix 2 figure 5: Estimated associations between type of health insurance and unmet need by countries: Odds ratio (OR) and adjusted odds ratio (aOR) 9

Appendix 2figure 6: Estimated associations between type of health insurance and mental care by countries: Odds ratio (OR) and adjusted odds ratio (aOR) 10

Appendix 2 figure 7: Estimated associations between type of health insurance and cancer screening by countries: Odds ratio (OR) and adjusted odds ratio (aOR) 11

Appendix 2 figure 8: Estimated associations between type of health insurance and cardiovascular examination by countries: Odds ratio (OR) and adjusted odds ratio (aOR) ...12

Appendix 2 figure 9: Estimated associations between type of health insurance and eye or teeth examination by countries: Odds ratio (OR) and adjusted odds ratio (aOR) 13

Appendix 2 figure 10: Estimated associations between type of health insurance and confidence in ability to receive and afford care by countries: Odds ratio (OR) and adjusted odds ratio (aOR) 14

Appendix 2: Supplementary Methodology and Results

Appendix 2 table 1: Survey questions on healthcare utilization outcomes and insurance

Outcome	Question	Response
**Insurance status**	Do you have health insurance?	Yes/No
**Insurance type**	What type of health insurance do you have?	Public/Private
**Number of visits to a facility in the past year**	How many healthcare visits in total have you made in the past 12 months?	Numerical
	Could you please tell me if the total number of healthcare visits you have made in the past 12 months is…?	1. 0<br>2. 1-4<br>3. 5-9<br>4. 10 or more
**Unmet need in the past year**	In the last twelve months, was there a time when you had a health problem and needed medical attention, but you did not get healthcare from a provider?	Yes/No
**Received mental care in the past year**	Can you please tell me if you have received any of the following health services in the past 12 months from any healthcare provider?<br>Received care for depression, anxiety, or another mental health condition. This may include feeling sad or worried a lot of the time.	Yes/No
**Received mammogram in the past year**	Can you please tell me if you have received any of the following health services in the past 12 months from any healthcare provider?<br>Received a mammogram? (Respondents who identify as female ages 50 and older)	Yes/No
**Received cervical cancer screening in the past year**	Can you please tell me if you have received any of the following health services in the past 12 months from any healthcare provider?<br>Received cervical cancer screening, like a pap test or visual inspection? (Respondents who identify as female over age 18)	Yes/No
**Had a blood pressure test in the past year**	Can you please tell me if you have received any of the following health services in the past 12 months from any healthcare provider?<br>Had your blood pressure tested?	Yes/No
**Had a blood sugar test in the past year**	Can you please tell me if you have received any of the following health services in the past 12 months from any healthcare provider?<br>Had a blood sugar test?	Yes/No
**Had a blood cholesterol test in the past year**	Can you please tell me if you have received any of the following health services in the past 12 months from any healthcare provider?<br>Had a blood cholesterol test?	Yes/No
**Had eye or vision checked in the past year**	Can you please tell me if you have received any of the following health services in the past 12 months from any healthcare provider?<br>Had your eyes or vision checked	Yes/No
**Had your teeth checked in the past year**	Can you please tell me if you have received any of the following health services in the past 12 months from any healthcare provider?<br>Had your teeth checked	Yes/No
**Confidence in receiving care**	Now we would like to hear your thoughts on the healthcare system in your country as a whole, including public, private, and NGO healthcare facilities and providers. How confident are you…<br>That you would receive good quality healthcare if you became very sick?	1. Very confident<br>2. Somewhat confident<br>3. Not too confident<br>4. Not at all confident
**Confidence in ability to afford health care**	Now we would like to hear your thoughts on the healthcare system in your country, including public, private, and NGO healthcare facilities and providers.<br>How confident are you…<br>That you would be able to afford the healthcare you needed if you became very sick?	1. Very confident<br>2. Somewhat confident<br>3. Not too confident<br>4. Not at all confident

Appendix 2 figure 1: Anderson health utilization conceptual framework

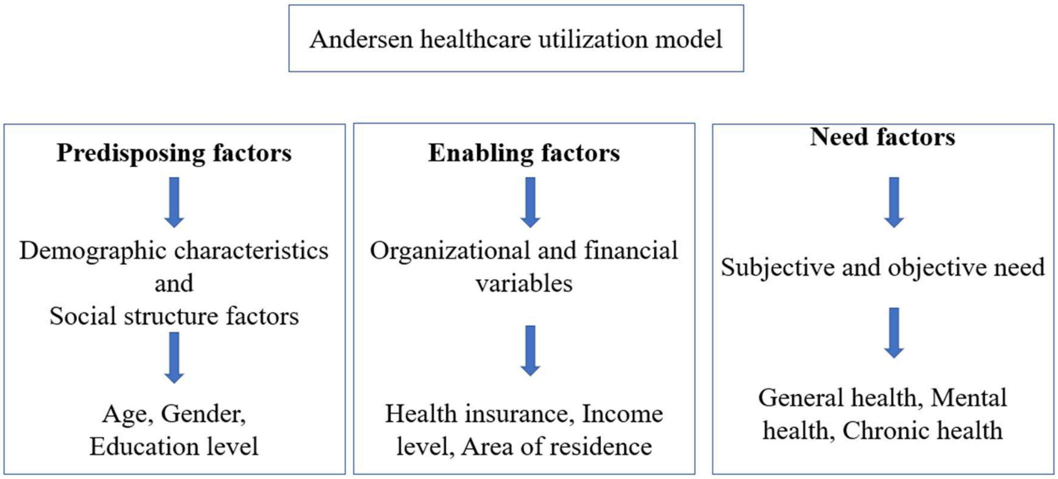

Appendix 2 table 2: Descriptive characteristics by country

CHARACTERISTICS	ETHIOPIA N = 2549	KENYA N = 2013	SOUTH AFRICA N = 1956	LAOS N = 1759	INDIA N = 1497	POOLED N = 9774
**Enabling factors**						
**Insurance**						
Private+	37 (1%)	68 (3%)	253 (13%)	220 (13%)	110 (7%)	689 (7%)
Public	1,536 (60%)	588 (29%)	0 (0%)	1,539 (87%)	237 (16%)	3,901 (40%)
Uninsured	975 (38%)	1,357 (67%)	1,702 (87%)	0 (0%)	1,150 (77%)	5,184 (53%)
**Area of residence**						
Rural	1,794 (70%)	1,313 (65%)	609 (31%)	1,181 (67%)	753 (50%)	5,650 (58%)
Urban	755 (30%)	700 (35%)	1,347 (69%)	578 (33%)	744 (50%)	4,123 (42%)
**Household income**						
Low/Middle	1,747 (69%)	1,413 (70%)	1,451 (74%)	883 (50%)	1,297 (87%)	6,791 (69%)
Highest	801 (31%)	600 (30%)	505 (26%)	876 (50%)	200 (13%)	2,983 (31%)
**Predisposing factors**						
**Age**						
18-29	1,102 (43%)	819 (41%)	560 (29%)	548 (31%)	544 (36%)	3,573 (37%)
30-39	625 (25%)	502 (25%)	521 (27%)	358 (20%)	305 (20%)	2,311 (24%)
40-49	396 (16%)	359 (18%)	393 (20%)	398 (23%)	322 (22%)	1,867 (19%)
50-59	189 (7%)	192 (10%)	208 (11%)	298 (17%)	156 (10%)	1,041 (11%)
60 or older	237 (9%)	142 (7%)	274 (14%)	158 (9%)	171 (11%)	982 (10%)
**Gender**						
Male	1,304 (51%)	1,013 (50%)	944 (48%)	878 (50%)	831 (56%)	4,969 (51%)
Female	1,245 (49%)	1,000 (50%)	1,012 (52%)	882 (50%)	666 (44%)	4,805 (49%)
**Education**						
None/Primary	2,021 (79%)	1,268 (63%)	1,091 (56%)	933 (53%)	502 (34%)	5,815 (60%)
Secondary	312 (12%)	520 (26%)	643 (33%)	548 (31%)	416 (28%)	2,438 (25%)
Post-secondary	215 (8%)	225 (11%)	222 (11%)	279 (16%)	579 (39%)	1,520 (16%)
**Need factors**						
**General health**						
Poor or fair	766 (30%)	373 (19%)	605 (31%)	503 (29%)	611 (41%)	2,858 (29%)
Good or higher	1,782 (70%)	1,640 (81%)	1,351 (69%)	1,256 (71%)	886 (59%)	6,915 (71%)
**Mental health**						
Poor or fair	548 (22%)	159 (8%)	383 (20%)	409 (23%)	508 (34%)	2,007 (21%)
Good or higher	2,000 (78%)	1,854 (92%)	1,573 (80%)	1,350 (77%)	989 (66%)	7,767 (79%)
**Chronic health condition**						
No	2,207 (87%)	1,680 (83%)	1,414 (72%)	1,336 (76%)	1,288 (86%)	7,926 (81%)
Yes	341 (13%)	333 (17%)	542 (28%)	423 (24%)	209 (14%)	1,847 (19%)

Appendix 2 table 3: Healthcare utilization by health insurance type and socio-demographic characteristics

Characteristics	Visits in the past 12 mo. N = 6542 (67%)	Unmet need N = 1319 (14%)	Mental healthcare N = 638 (7%)	Cancer screening N = 782 (16%)	Cardio. exam N = 5201 (53%)	Eye or teeth exam N = 2629 (27%)	Confident receive & afford care N = 5356 (55%)
**Enabling factors**							
**Insurance**							
Private+	524 (76%)	92 (13%)	63 (9%)	64 (19%)	484 (70%)	321 (47%)	444 (66%)
Public	2,520 (65%)	586 (15%)	166 (4%)	267 (15%)	1,991 (51%)	935 (24%)	2,283 (59%)
Uninsured	3,498 (68%)	641 (12%)	409 (8%)	450 (17%)	2,726 (53%)	1,373 (27%)	2,629 (51%)
**Area of residence**							
Rural	3,768 (67%)	878 (16%)	281 (5%)	369 (13%)	2,707 (48%)	1,249 (22%)	3,143 (56%)
Urban	2,775 (68%)	441 (11%)	357 (9%)	413 (21%)	2,495 (61%)	1,379 (34%)	2,213 (54%)
**Household income**							
Low/Middle	4,519 (67%)	984 (15%)	481 (7%)	582 (17%)	3,453 (51%)	1,693 (25%)	3,561 (53%)
Highest	2,024 (68%)	336 (11%)	157 (5%)	199 (16%)	1,748 (59%)	935 (31%)	1,795 (61%)
**Predisposing factors**							
**Age**							
18-29	2,215 (62%)	605 (17%)	191 (5%)	221 (12%)	1,538 (43%)	776 (22%)	1,864 (53%)
30-39	1,606 (70%)	251 (11%)	155 (7%)	212 (18%)	1,220 (53%)	608 (26%)	1,231 (54%)
40-49	1,277 (69%)	231 (12%)	128 (7%)	155 (17%)	1,137 (61%)	537 (29%)	1,085 (58%)
50-59	756 (73%)	120 (12%)	71 (7%)	89 (19%)	660 (63%)	348 (33%)	616 (60%)
60 or older	688 (70%)	112 (11%)	93 (10%)	106 (25%)	647 (66%)	360 (37%)	560 (57%)
**Gender**							
Male	3,083 (62%)	592 (12%)	290 (6%)		2,421 (49%)	1,366 (28%)	2,750 (56%)
Female	3,459 (72%)	727 (15%)	348 (7%)		2,780 (58%)	1,263 (26%)	2,605 (55%)
**Education**							
None/Primary	3,938 (68%)	864 (15%)	390 (7%)	527 (17%)	3,011 (52%)	1,437 (25%)	3,070 (53%)
Secondary	1,626 (67%)	294 (12%)	153 (6%)	173 (16%)	1,318 (54%)	697 (29%)	1,383 (57%)
Post-secondary	979 (65%)	161 (11%)	95 (6%)	81 (12%)	873 (57%)	494 (33%)	902 (60%)
**Need factors**							
**General health**							
Poor or fair	2,119 (74%)	452 (16%)	276 (10%)	336 (23%)	1,696 (59%)	827 (29%)	1,535 (54%)
Good or higher	4,423 (64%)	868 (13%)	362 (5%)	445 (13%)	3,505 (51%)	1,801 (26%)	3,820 (56%)
**Mental health**							
Poor or fair	1,355 (68%)	270 (13%)	230 (12%)	205 (20%)	1,090 (54%)	563 (28%)	1,098 (56%)
Good or higher	5,188 (67%)	1,049 (14%)	408 (5%)	577 (16%)	4,111 (53%)	2,065 (27%)	4,258 (55%)
**Chronic health condition**							
No	4,920 (62%)	960 (12%)	430 (5%)	506 (14%)	3,759 (47%)	1,978 (25%)	4,398 (56%)
Yes	1,623 (88%)	360 (19%)	208 (11%)	275 (27%)	1,442 (78%)	651 (35%)	958 (52%)

Outcomes included visits in the past 12months, unmet need, mental healthcare, cancer screening, cardiovascular examination, eye or teeth examination and the ability to receive and afford care. Cardiovascular examination is a combination of blood pressure, blood sugar and cholesterol checks. The summary provided for healthcare utilization indicates users of healthcare facility in past 12 months, confidence in ability to receive and afford care, those who agree to having an unmet need, receiving mental healthcare, cancer screening, cardiovascular and eye or teeth examination.

Appendix 2 figure 2: Source of care facility type by insurance coverage

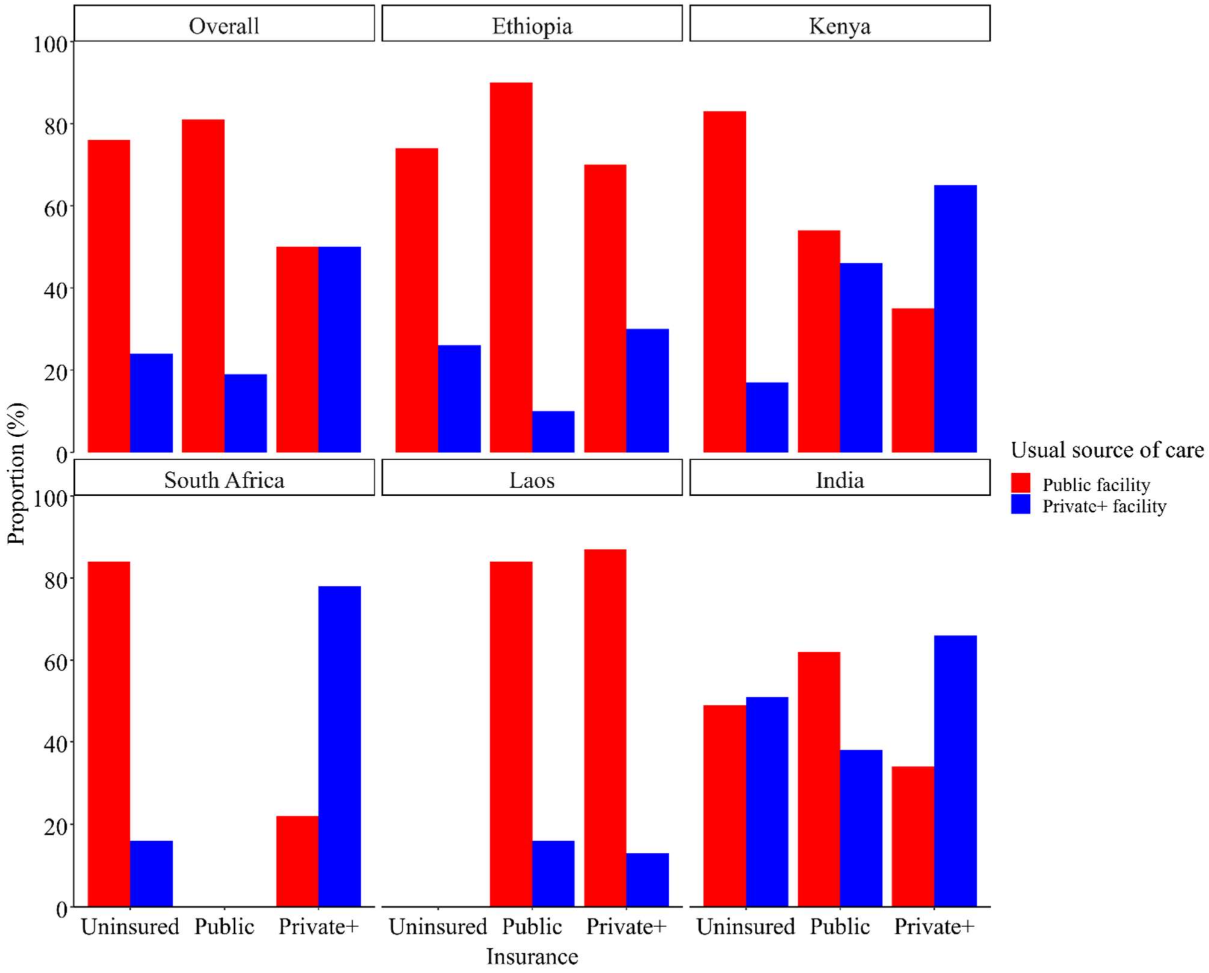

Appendix 2 figure 3: Main reason for choice of usual source of care per country

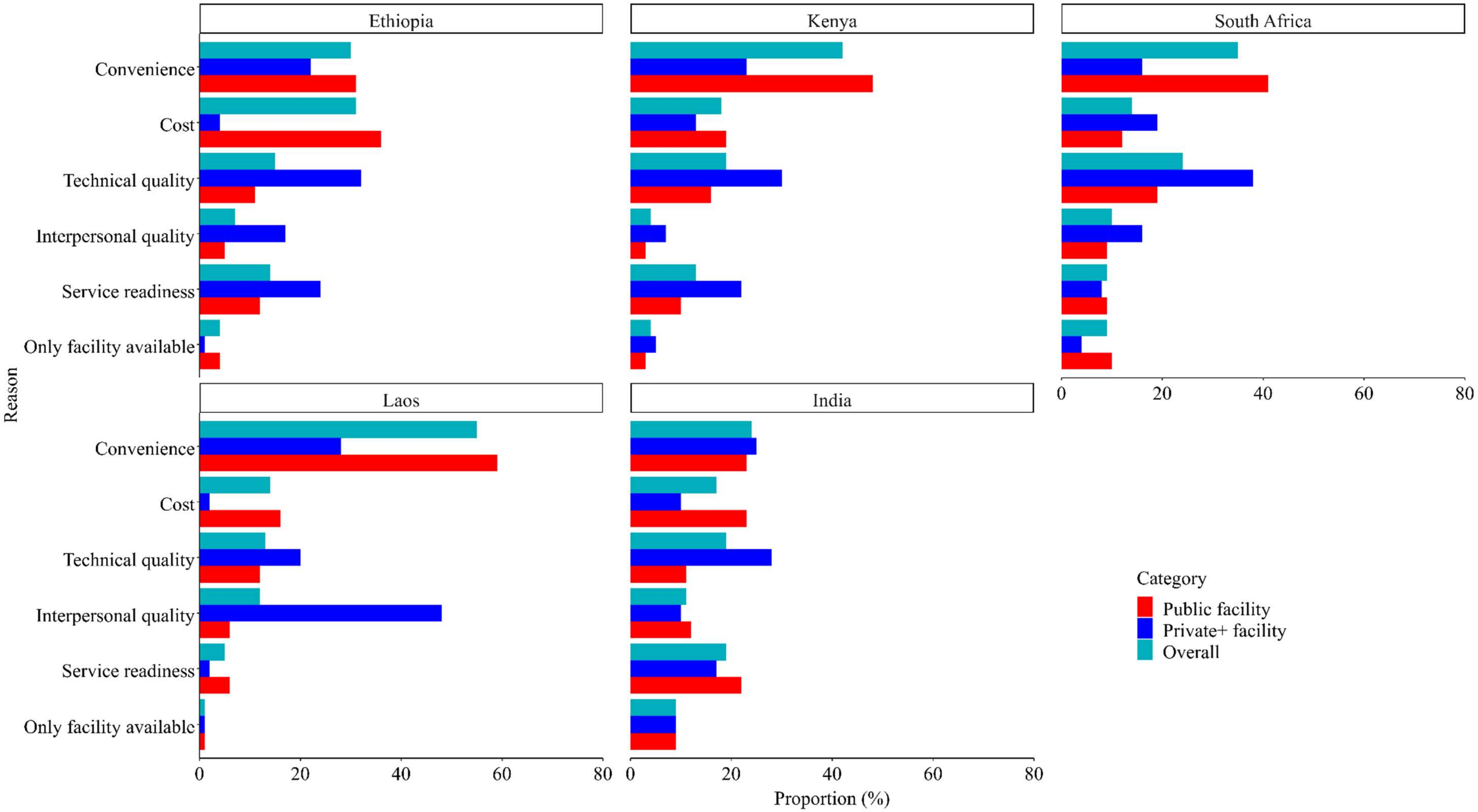

Appendix 2 figure 4: Estimated associations between type of health insurance and health care visits by countries: Odds ratio (OR) and adjusted odds ratio (aOR)

	ETHIOPIA		KENYA		SOUTH AFRICA		LAOS		INDIA	
FACTORS	OR (95%CI)	aOR(95%CI)	OR (95%CI)	aOR(95%CI)	OR (95%CI)	aOR(95%CI)	OR (95%CI)	aOR(95%CI)	OR (95%CI)	aOR(95%CI)
**Enabling factors**										
**Insurance**										
Private+										
Public	0.53 (0.15, 1.81)	0.64 (0.22, 1.84)	0.38 (0.17, 0.84)	0.38 (0.17, 0.85)			0.65 (0.38, 1.11)	0.74 (0.43, 1.29)	1.34 (0.61, 2.93)	1.68 (0.77, 3.67)
Uninsured	0.53 (0.16, 1.81)	0.52 (0.19, 1.45)	0.58 (0.27, 1.26)	0.41 (0.19, 0.91)	0.71 (0.45, 1.13)	0.69 (0.41, 1.16)			0.51 (0.27, 0.98)	0.55 (0.30, 1.01)
**Area of residence**										
Rural										
Urban	1.48 (1.12, 1.95)		0.42 (0.23, 0.78)	0.58 (0.33, 1.05)	1.1 (0.77, 1.55)		1.11 (0.81, 1.50)		0.94 (0.67, 1.31)	
**Household income**										
Low/Middle										
Highest	1.53 (0.96, 2.44)	1.63 (1.00, 2.68)	0.55 (0.37, 0.82)		1 (0.72, 1.38)		1.22 (0.86, 1.73)	1.24 (0.85, 1.79)	0.77 (0.41, 1.43)	0.68 (0.42, 1.10)
**Predisposing factors**										
**Age**										
18-29										
30-39	2 (1.20, 3.35)	1.71 (1.06, 2.77)	0.97 (0.66, 1.41)	0.75 (0.52, 1.08)	1.33 (0.94, 1.89)	1.16 (0.81, 1.66)	1.28 (0.78, 2.10)	1.27 (0.75, 2.14)	1.03 (0.76, 1.41)	0.8 (0.58, 1.11)
40-49	1.73 (1.10, 2.71)	1.47 (0.91, 2.38)	0.97 (0.60, 1.56)	0.8 (0.48, 1.34)	1.31 (0.84, 2.03)	0.95 (0.60, 1.50)	1.19 (0.75, 1.88)	1.09 (0.65, 1.82)	1.25 (0.82, 1.91)	0.92 (0.58, 1.46)
50-59	2.16 (1.21, 3.86)	1.68 (0.90, 3.12)	2.47 (1.27, 4.79)	1.69 (0.90, 3.19)	1.9 (1.06, 3.41)	0.98 (0.51, 1.86)	0.83 (0.49, 1.43)	0.71 (0.40, 1.27)	2.05 (1.04, 4.06)	1.53 (0.78, 2.99)
60 or older	1.89 (0.71, 5.00)	1.38 (0.61, 3.13)	1.61 (0.69, 3.76)	0.78 (0.37, 1.67)	1.86 (0.92, 3.75)	0.71 (0.32, 1.56)	2.01 (0.93, 4.33)	1.57 (0.67, 3.71)	0.56 (0.24, 1.32)	0.41 (0.19, 0.86)
**Gender**										
Male										
Female	1.26 (0.92, 1.74)	1.32 (0.92, 1.90)	2.54 (1.87, 3.47)	2.2 (1.61, 3.00)	2.19 (1.58, 3.04)	1.88 (1.36, 2.60)	1.49 (1.05, 2.13)	1.7 (1.19, 2.44)	0.99 (0.72, 1.37)	
**Education**										
None/Primary										
Secondary	1.3 (0.95, 1.76)	1.5 (1.18, 1.92)	0.41 (0.28, 0.60)	0.54 (0.34, 0.85)	1 (0.73, 1.39)		1.07 (0.75, 1.52)	1.3 (0.86, 1.94)	1.19 (0.75, 1.87)	
Post-secondary	1.55 (1.17, 2.06)	1.52 (1.06, 2.18)	0.57 (0.37, 0.89)	0.87 (0.54, 1.40)	0.78 (0.55, 1.09)		1.36 (0.95, 1.93)	1.5 (1.0, 2.26)	1.12 (0.75, 1.67)	
**Need factors**										
**General health**										
Poor or fair										
Good or higher	0.43 (0.25, 0.72)	0.48 (0.25, 0.92)	0.44 (0.26, 0.74)		0.45 (0.30, 0.69)	0.63 (0.40, 1.01)	0.8 (0.54, 1.19)	0.75 (0.46, 1.23)	0.67 (0.48, 0.94)	0.72 (0.49, 1.07)
**Mental health**										
Poor or fair										
Good or higher	0.75 (0.49, 1.17)	1.2 (0.71, 2.01)	0.44 (0.22, 0.87)	0.66 (0.33, 1.30)	0.62 (0.39, 0.99)		1.17 (0.79, 1.73)	1.67 (1.04, 2.66)	0.93 (0.66, 1.32)	1.29 (0.86, 1.93)
**Chronic health condition**										
No										
Yes	7.28 (4.54, 11.7)	5.63 (3.33, 9.54)	8.94 (4.49, 17.8)	7.33 (3.74, 14.4)	5.48 (3.16, 9.50)	4.6 (2.58, 8.21)	2.28 (1.48, 3.50)	2.46 (1.47, 4.12)	3.78 (1.88, 7.60)	3.66 (1.83, 7.32)

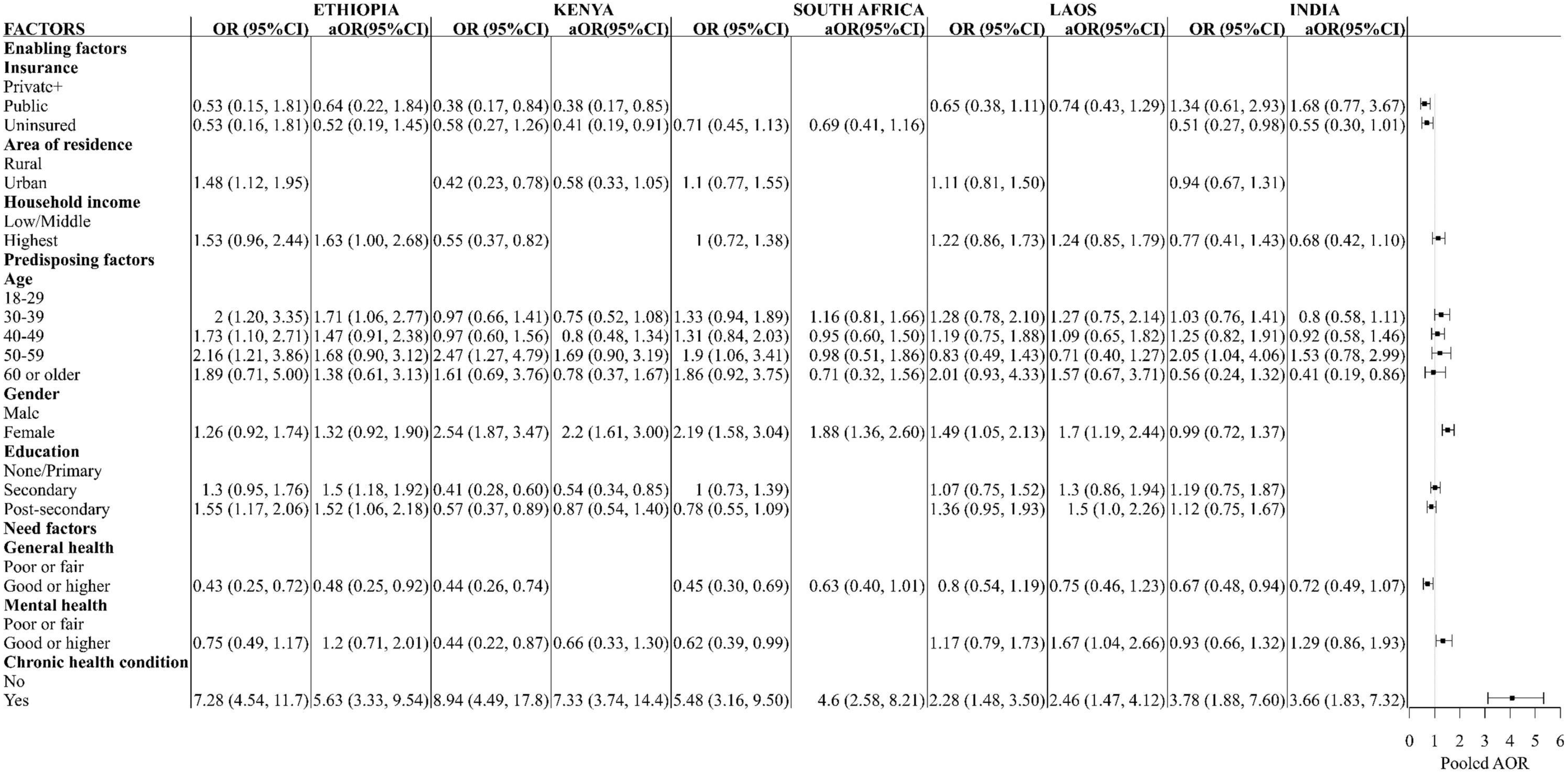

Appendix 2 figure 5: Estimated associations between type of health insurance and unmet need by countries: Odds ratio (OR) and adjusted odds ratio (aOR)

	ETHIOPIA		KENYA		SOUTH AFRICA		LAOS		INDIA	
**Factors**	**OR (95%CI)**	**aOR(95%CI)**	**OR (95%CI)**	**aOR(95%CI)**	**OR (95%CI)**	**aOR(95%CI)**	**OR (95%CI)**	**aOR(95%CI)**	**OR (95%CI)**	**aOR(95%CI)**
**Enabling factors**										
**Insurance**										
Private+										
Public	1.02 (0.34, 3.03)	0.59 (0.18, 1.97)	0.97 (0.45, 2.09)	0.69 (0.31, 1.55)			0.62 (0.37, 1.03)	0.63 (0.37, 1.09)	4.68 (1.31, 16.8)	4.36 (1.24, 15.3)
Uninsured	0.89 (0.35, 2.28)	0.58 (0.18, 1.85)	1.1 (0.54, 2.26)	0.51 (0.25, 1.05)	1.61 (0.70, 3.69)	1.3 (0.60, 2.80)			2.33 (0.85, 6.39)	2.29 (0.77, 6.81)
**Area of residence**										
Rural										
Urban	0.47 (0.27, 0.79)	0.63 (0.40, 0.99)	0.7 (0.39, 1.24)		0.72 (0.45, 1.15)	0.73 (0.45, 1.20)	1.24 (0.84, 1.84)		0.65 (0.35, 1.24)	
**Household income**										
Low/Middle										
Highest	0.52 (0.27, 0.99)	0.61 (0.34, 1.09)	0.44 (0.28, 0.69)	0.55 (0.36, 0.84)	0.68 (0.42, 1.13)	0.71 (0.44, 1.14)	0.92 (0.60, 1.42)		0.75 (0.28, 2.02)	
**Predisposing factors**										
**Age**										
18-29										
30-39	0.39 (0.18, 0.86)	0.34 (0.17, 0.69)	0.66 (0.36, 1.21)	0.47 (0.23, 0.95)	0.97 (0.60, 1.58)	0.89 (0.54, 1.45)	0.36 (0.21, 0.62)	0.35 (0.20, 0.62)	1.61 (0.87, 2.97)	1.31 (0.66, 2.59)
40-49	0.55 (0.20, 1.54)	0.34 (0.13, 0.90)	0.82 (0.36, 1.88)	0.61 (0.28, 1.32)	0.53 (0.26, 1.07)	0.43 (0.20, 0.92)	0.75 (0.43, 1.28)	0.75 (0.43, 1.32)	0.92 (0.43, 1.97)	0.74 (0.32, 1.70)
50-59	0.28 (0.11, 0.72)	0.15 (0.05, 0.49)	1.1 (0.49, 2.50)	0.73 (0.30, 1.81)	0.86 (0.41, 1.83)	0.6 (0.27, 1.31)	0.37 (0.18, 0.74)	0.4 (0.19, 0.82)	0.86 (0.21, 3.55)	0.76 (0.16, 3.56)
60 or older	0.57 (0.10, 3.43)	0.23 (0.05, 0.97)	1.35 (0.49, 3.74)	0.57 (0.24, 1.38)	0.41 (0.15, 1.16)	0.26 (0.10, 0.72)	0.44 (0.14, 1.37)	0.45 (0.14, 1.49)	1.37 (0.26, 7.19)	1.09 (0.29, 4.14)
**Gender**										
Male										
Female	1.19 (0.42, 3.36)		1.28 (0.75, 2.19)		1.11 (0.72, 1.71)		1.75 (1.16, 2.63)	1.81 (1.21, 2.69)	1.05 (0.55, 2.00)	
**Education**										
None/Primary										
Secondary	0.49 (0.25, 0.96)	0.49 (0.25, 0.94)	0.58 (0.38, 0.87)	0.61 (0.38, 0.99)	1.22 (0.78, 1.91)	1.38 (0.85, 2.25)	1.16 (0.73, 1.86)	1.05 (0.65, 1.71)	0.76 (0.31, 1.88)	
Post-secondary	0.43 (0.25, 0.73)	0.82 (0.51, 1.30)	0.43 (0.28, 0.67)	0.48 (0.29, 0.80)	1.17 (0.71, 1.92)	1.59 (0.87, 2.88)	1.53 (0.96, 2.45)	1.46 (0.89, 2.39)	1.03 (0.45, 2.32)	
**Need factors**										
**General health**										
Poor or fair										
Good or higher	0.51 (0.17, 1.58)	0.64 (0.25, 1.66)	0.46 (0.32, 0.66)	0.53 (0.39, 0.73)	0.72 (0.45, 1.15)	0.66 (0.38, 1.13)	1.13 (0.68, 1.88)		0.59 (0.31, 1.16)	0.65 (0.29, 1.47)
**Mental health**										
Poor or fair										
Good or higher	0.57 (0.22, 1.49)	0.66 (0.36, 1.21)	1.26 (0.55, 2.86)	2.35 (1.29, 4.28)	0.74 (0.44, 1.24)		1 (0.60, 1.68)		0.74 (0.39, 1.40)	
**Chronic health condition**										
No										
Yes	2.03 (0.66, 6.23)	2.93 (1.25, 6.88)	3.15 (2.30, 4.32)	2.69 (2.04, 3.53)	1.52 (0.96, 2.43)	2.04 (1.27, 3.27)	0.92 (0.56, 1.52)		1.94 (0.70, 5.36)	1.78 (0.53, 5.99)

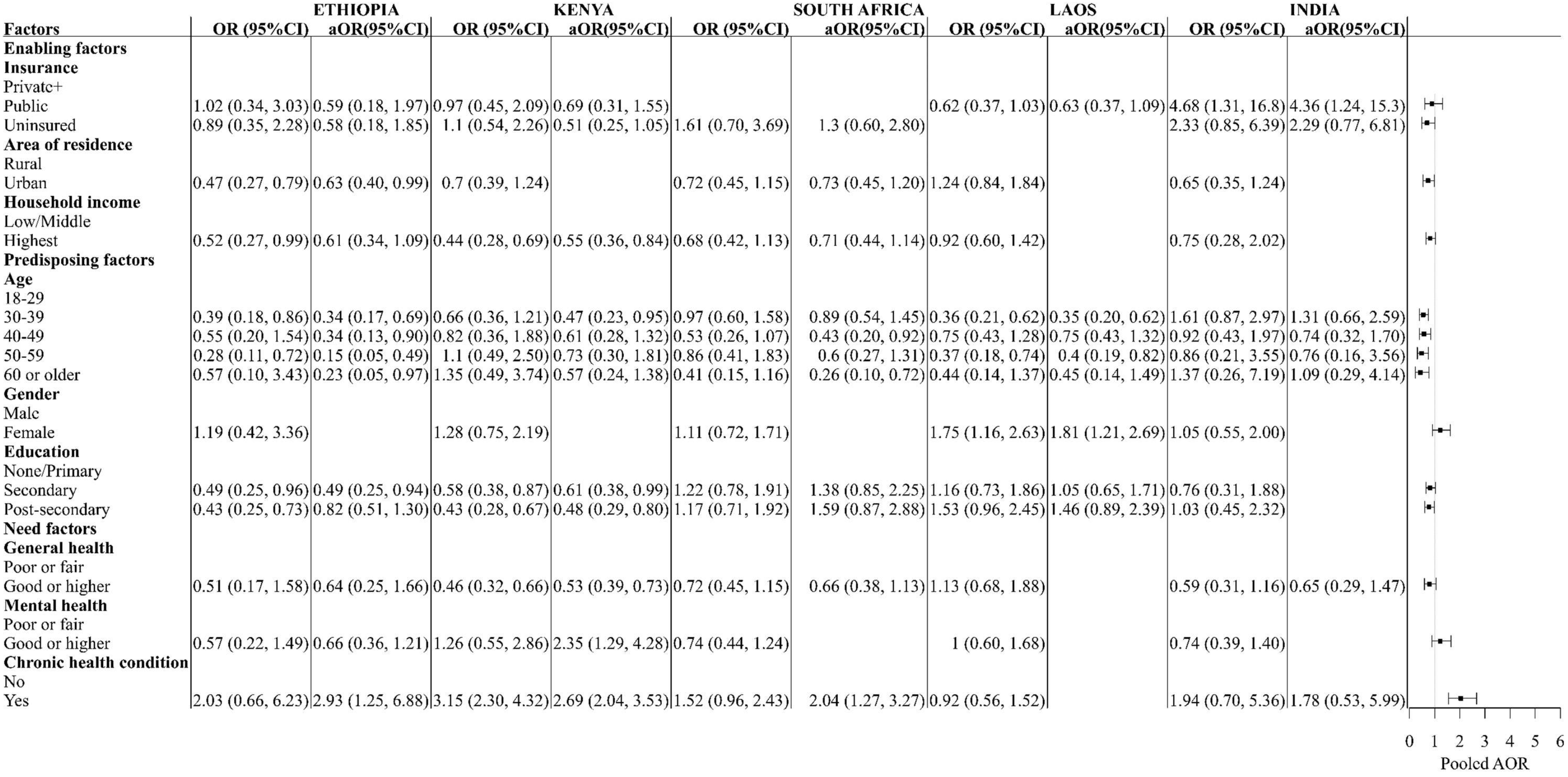

Appendix 2figure 6: Estimated associations between type of health insurance and mental care by countries: Odds ratio (OR) and adjusted odds ratio (aOR)

Factors	ETHIOPIA OR (95%CI)	ETHIOPIA aOR(95%CI)	KENYA OR (95%CI)	KENYA aOR(95%CI)	SOUTH AFRICA OR (95%CI)	SOUTH AFRICA aOR(95%CI)	LAOS OR (95%CI)	LAOS aOR(95%CI)	INDIA OR (95%CI)	INDIA aOR(95%CI)
**Enabling factors**										
**Insurance**										
Private+										
Public	0.88 (0.27, 2.89)	0.92 (0.27, 3.09)	1.02 (0.42, 2.51)	0.92 (0.38, 2.26)			0.47 (0.14, 1.60)	0.65 (0.17, 2.48)	2.53 (0.94, 6.81)	2.8 (0.97, 8.07)
Uninsured	1.02 (0.30, 3.48)	1.11 (0.33, 3.71)	0.94 (0.37, 2.40)	0.79 (0.31, 2.06)	0.67 (0.39, 1.15)	0.6 (0.32, 1.13)			0.88 (0.39, 2.00)	0.82 (0.31, 2.14)
**Area of residence**										
Rural										
Urban	1.26 (0.67, 2.37)		1.14 (0.53, 2.45)		1.59 (0.93, 2.71)	1.35 (0.78, 2.33)	1.12 (0.32, 4.00)	0.5 (0.12, 2.13)	1.02 (0.57, 1.82)	
**Household income**										
Low/Middle										
Highest	0.77 (0.42, 1.42)		1.23 (0.59, 2.58)		1.06 (0.65, 1.74)		26.4 (5.13, 136)	34.7 (4.99, 241)	0.32 (0.11, 0.91)	0.41 (0.15, 1.11)
**Predisposing factors**										
**Age**										
18-29										
30-39	2.15 (0.59, 7.87)	2.13 (0.61, 7.47)	0.47 (0.17, 1.28)	0.44 (0.17, 1.15)	1.15 (0.69, 1.94)	1.01 (0.58, 1.76)	1.24 (0.17, 8.78)	0.94 (0.12, 7.21)	0.91 (0.48, 1.74)	0.63 (0.33, 1.22)
40-49	2.19 (0.72, 6.68)	2.13 (0.70, 6.49)	1.25 (0.40, 3.89)	1.12 (0.36, 3.44)	0.77 (0.45, 1.31)	0.51 (0.28, 0.91)	7.5 (1.10, 51.0)	6.26 (0.72, 54.1)	1.18 (0.54, 2.59)	0.83 (0.36, 1.89)
50-59	2.51 (0.54, 11.6)	2.37 (0.57, 9.91)	0.81 (0.27, 2.47)	0.68 (0.22, 2.14)	1.62 (0.82, 3.19)	0.78 (0.37, 1.66)	0.89 (0.12, 6.43)	0.49 (0.05, 4.44)	0.71 (0.24, 2.07)	0.68 (0.22, 2.11)
60 or older	3.24 (0.38, 27.7)	2.84 (0.40, 20.1)	0.13 (0.02, 1.07)	0.09 (0.01, 0.79)	2.37 (0.98, 5.74)	1.03 (0.46, 2.30)	4.45 (0.50, 39.6)	2.33 (0.20, 27.5)	0 (0.00, 0.00)	0 (0.00, 0.00)
**Gender**										
Male										
Female	0.75 (0.44, 1.30)	0.78 (0.43, 1.39)	0.52 (0.20, 1.34)	0.52 (0.20, 1.33)	2.01 (1.26, 3.21)	1.59 (1.02, 2.47)	1.27 (0.31, 5.15)	1.59 (0.47, 5.44)	1.58 (0.89, 2.81)	1.48 (0.82, 2.66)
**Education**										
None/Primary										
Secondary	0.91 (0.46, 1.83)		1.52 (0.67, 3.49)		0.57 (0.36, 0.91)	0.66 (0.42, 1.05)	1.7 (0.19, 15.0)		1.4 (0.67, 2.91)	1.24 (0.61, 2.52)
Post-secondary	0.67 (0.38, 1.20)		1.68 (0.74, 3.82)		0.87 (0.52, 1.47)	0.95 (0.54, 1.67)	3.49 (0.45, 26.8)		0.8 (0.42, 1.53)	0.71 (0.36, 1.40)
**Need factors**										
**General health**										
Poor or fair										
Good or higher	0.7 (0.35, 1.37)		0.65 (0.22, 1.94)	0.49 (0.18, 1.34)	0.42 (0.25, 0.71)		0.42 (0.09, 1.93)		0.72 (0.41, 1.29)	
**Mental health**										
Poor or fair										
Good or higher	0.64 (0.25, 1.65)	0.74 (0.38, 1.45)	1.03 (0.30, 3.50)		0.27 (0.15, 0.47)	0.3 (0.18, 0.50)	0.55 (0.08, 3.79)		0.58 (0.32, 1.03)	0.62 (0.35, 1.10)
**Chronic health condition**										
No										
Yes	1.35 (0.59, 3.09)		1.46 (0.48, 4.41)		3.09 (1.84, 5.21)	2.32 (1.45, 3.70)	3.68 (0.88, 15.4)	3.89 (0.79, 19.2)	1.63 (0.76, 3.50)	1.96 (0.94, 4.09)

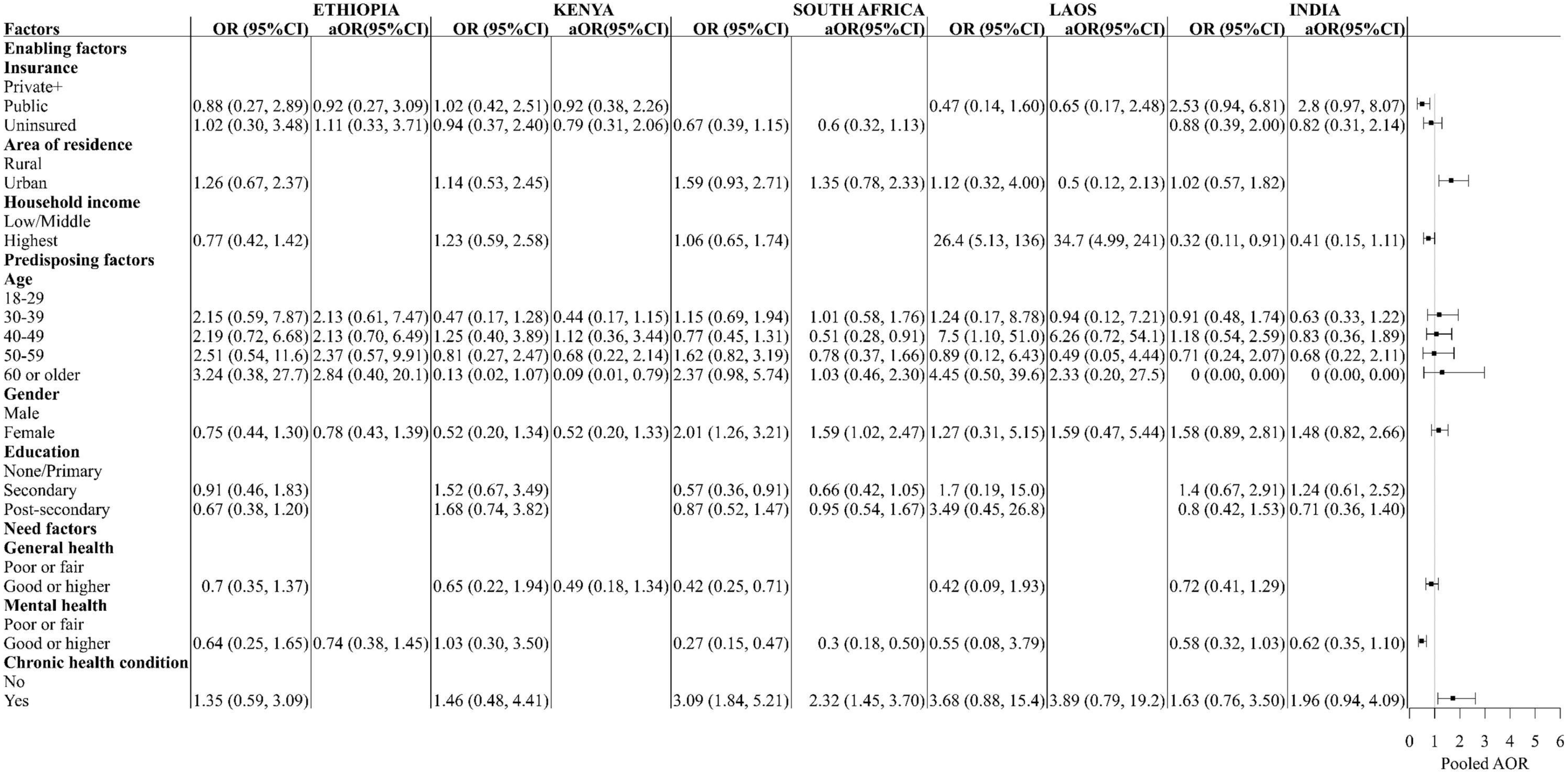

Appendix 2 figure 7: Estimated associations between type of health insurance and cancer screening by countries: Odds ratio (OR) and adjusted odds ratio (aOR)

	ETHIOPIA		KENYA		SOUTH AFRICA		LAOS		INDIA	
**Factors**	**OR (95%CI)**	**aOR(95%CI)**	**OR (95%CI)**	**aOR(95%CI)**	**OR (95%CI)**	**aOR(95%CI)**	**OR (95%CI)**	**aOR(95%CI)**	**OR (95%CI)**	**aOR(95%CI)**
**Enabling factors**										
**Insurance**										
Private+										
Public	1.34 (0.39, 4.61)	3.08 (0.77, 12.3)	0.56 (0.19, 1.61)	0.5 (0.15, 1.62)			0.76 (0.41, 1.40)	0.56 (0.31, 1.01)	4.49 (0.91, 22.1)	2 (0.41, 9.73)
Uninsured	2.29 (0.67, 7.82)	4.04 (1.09, 14.9)	0.27 (0.09, 0.81)	0.19 (0.05, 0.69)	1.51 (0.83, 2.76)	1.17 (0.59, 2.32)			1.33 (0.34, 5.25)	0.49 (0.11, 2.19)
**Area of residence**										
Rural										
Urban	2.1 (1.09, 4.04)	2.45 (1.17, 5.13)	0.94 (0.49, 1.82)		1.61 (0.93, 2.79)	1.53 (0.88, 2.64)	1.75 (1.01, 3.04)		0.82 (0.36, 1.87)	
**Household income**										
Low/Middle										
Highest	1.22 (0.47, 3.13)		1.17 (0.61, 2.24)		0.52 (0.31, 0.87)	0.64 (0.37, 1.12)	1.11 (0.61, 1.99)		0.26 (0.09, 0.78)	
**Predisposing factors**										
**Age**										
18-29										
30-39	1.09 (0.37, 3.17)	0.86 (0.27, 2.76)	0.94 (0.41, 2.18)	0.6 (0.26, 1.40)	2.51 (1.40, 4.47)	2.3 (1.26, 4.21)	1.45 (0.57, 3.67)	1.64 (0.62, 4.37)	1.01 (0.39, 2.62)	0.72 (0.28, 1.86)
40-49	1.33 (0.40, 4.47)	1.21 (0.36, 4.03)	1.73 (0.82, 3.66)	1.21 (0.56, 2.62)	1.66 (0.83, 3.33)	1.07 (0.49, 2.34)	1.69 (0.72, 3.95)	2.18 (0.88, 5.45)	0.41 (0.13, 1.27)	0.23 (0.06, 0.89)
50-59	0.36 (0.13, 1.00)	0.3 (0.09, 0.97)	2.44 (0.99, 6.06)	1.31 (0.44, 3.86)	2.53 (1.21, 5.30)	1.58 (0.72, 3.51)	1.4 (0.56, 3.52)	1.74 (0.67, 4.53)	0 (0.00, 0.00)	0 (0.00, 0.00)
60 or older	0.04 (0.00, 0.31)	0.03 (0.00, 0.30)	5.82 (0.97, 35.0)	2.94 (0.56, 15.5)	2.72 (1.03, 7.21)	1.48 (0.65, 3.37)	0.87 (0.31, 2.47)	1.51 (0.50, 4.57)	2.88 (0.40, 20.6)	3.57 (0.50, 25.8)
**Education**										
None/Primary										
Secondary	0.96 (0.50, 1.84)	0.53 (0.28, 1.01)	0.57 (0.32, 1.00)	0.55 (0.25, 1.24)	0.54 (0.33, 0.88)		1.47 (0.82, 2.63)	1.81 (0.92, 3.56)	2.3 (0.79, 6.69)	2.71 (0.83, 8.86)
Post-secondary	1.43 (0.84, 2.44)	0.59 (0.32, 1.09)	1.15 (0.64, 2.08)	0.93 (0.39, 2.22)	0.49 (0.29, 0.85)		1.44 (0.79, 2.63)	1.64 (0.86, 3.12)	0.6 (0.24, 1.51)	0.6 (0.19, 1.87)
**Need factors**										
**General health**										
Poor or fair										
Good or higher	1.34 (0.77, 2.35)		0.26 (0.10, 0.67)	0.22 (0.09, 0.54)	0.32 (0.18, 0.55)	0.47 (0.27, 0.82)	0.56 (0.29, 1.07)		0.83 (0.36, 1.92)	
**Mental health**										
Poor or fair										
Good or higher	3.34 (1.27, 8.81)	3.08 (1.06, 8.95)	0.84 (0.21, 3.40)	3.13 (1.54, 6.37)	0.46 (0.23, 0.91)	0.68 (0.34, 1.34)	0.34 (0.18, 0.65)	0.29 (0.15, 0.57)	1.13 (0.47, 2.70)	
**Chronic health condition**										
No										
Yes	1.43 (0.75, 2.75)	1.89 (1.28, 2.79)	2.48 (1.26, 4.90)		2.27 (1.30, 3.94)	1.49 (0.91, 2.44)	1.65 (0.87, 3.11)		1.38 (0.38, 5.03)	

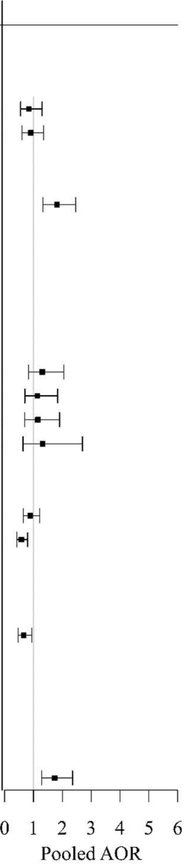

Appendix 2 figure 8: Estimated associations between type of health insurance and cardiovascular examination by countries: Odds ratio (OR) and adjusted odds ratio (aOR)

	ETHIOPIA		KENYA		SOUTH AFRICA		LAOS		INDIA	
**Factors**	**OR (95%CI)**	**aOR(95%CI)**	**OR (95%CI)**	**aOR(95%CI)**	**OR (95%CI)**	**aOR(95%CI)**	**OR (95%CI)**	**aOR(95%CI)**	**OR (95%CI)**	**aOR(95%CI)**
**Enabling factors**										
**Insurance**										
Private+										
Public	0.31 (0.11, 0.85)	0.7 (0.30, 1.65)	0.44 (0.21, 0.93)	0.57 (0.28, 1.15)			0.67 (0.40, 1.12)	0.83 (0.49, 1.40)	1.56 (0.73, 3.35)	1.78 (0.87, 3.66)
Uninsured	0.45 (0.18, 1.11)	0.71 (0.32, 1.56)	0.26 (0.13, 0.53)	0.3 (0.15, 0.61)	0.69 (0.46, 1.04)	0.8 (0.52, 1.21)			0.48 (0.25, 0.92)	0.54 (0.30, 0.99)
**Area of residence**										
Rural										
Urban	3.13 (1.98, 4.93)	2.46 (1.74, 3.48)	1.06 (0.68, 1.67)		1.52 (1.11, 2.10)	1.3 (0.93, 1.82)	1.16 (0.86, 1.57)		0.95 (0.68, 1.35)	
**Household income**										
Low/Middle										
Highest	1.91 (1.33, 2.75)	1.68 (1.22, 2.32)	1.1 (0.82, 1.48)	1.23 (0.92, 1.65)	1.02 (0.76, 1.38)		1.35 (0.96, 1.89)	1.23 (0.86, 1.77)	0.84 (0.45, 1.59)	0.65 (0.36, 1.16)
**Predisposing factors**										
**Age**										
18-29										
30-39	1.79 (1.07, 2.98)	1.35 (0.84, 2.15)	1.03 (0.78, 1.36)	0.76 (0.54, 1.06)	1.28 (0.91, 1.79)	1.13 (0.80, 1.59)	1.63 (1.01, 2.61)	1.63 (0.99, 2.67)	1.44 (1.04, 2.00)	1.13 (0.80, 1.59)
40-49	2.55 (1.31, 4.96)	2.28 (1.18, 4.40)	1.44 (0.84, 2.47)	1.31 (0.69, 2.50)	1.8 (1.20, 2.71)	1.36 (0.87, 2.13)	2.26 (1.43, 3.58)	2.17 (1.32, 3.55)	1.85 (1.20, 2.85)	1.39 (0.88, 2.22)
50-59	2.14 (0.94, 4.90)	1.66 (0.80, 3.47)	1.75 (0.93, 3.28)	1.42 (0.74, 2.72)	3.22 (1.81, 5.75)	1.82 (1.01, 3.27)	1.5 (0.87, 2.57)	1.37 (0.78, 2.42)	3.09 (1.55, 6.18)	2.59 (1.33, 5.04)
60 or older	2.26 (0.72, 7.05)	2.09 (0.67, 6.51)	2.75 (1.66, 4.57)	2.05 (0.97, 4.33)	3.33 (1.59, 6.96)	1.41 (0.65, 3.10)	3.1 (1.43, 6.73)	2.61 (1.12, 6.08)	1.91 (0.84, 4.34)	1.78 (0.82, 3.84)
**Gender**										
Male										
Female	0.88 (0.58, 1.33)		2.25 (1.64, 3.08)	2.64 (1.86, 3.76)	2.06 (1.53, 2.78)	1.69 (1.25, 2.29)	1.5 (1.06, 2.12)	1.75 (1.23, 2.50)	1.12 (0.80, 1.57)	1.47 (1.05, 2.08)
**Education**										
None/Primary										
Secondary	1.42 (0.80, 2.51)	1.19 (0.71, 2.01)	0.74 (0.51, 1.06)	0.82 (0.50, 1.35)	0.74 (0.54, 1.00)		0.85 (0.60, 1.21)	1.2 (0.81, 1.78)	1.08 (0.68, 1.71)	
Post-secondary	2.86 (1.87, 4.39)	1.6 (1.15, 2.23)	1.56 (1.11, 2.19)	1.54 (0.97, 2.44)	0.75 (0.54, 1.04)		1.6 (1.13, 2.27)	2.01 (1.33, 3.04)	1.07 (0.70, 1.62)	
**Need factors**										
**General health**										
Poor or fair										
Good or higher	0.59 (0.37, 0.95)	0.61 (0.37, 1.01)	0.58 (0.37, 0.93)		0.46 (0.32, 0.67)	0.77 (0.52, 1.16)	1.1 (0.75, 1.61)	1.45 (0.95, 2.21)	0.57 (0.40, 0.80)	0.67 (0.48, 0.95)
**Mental health**										
Poor or fair										
Good or higher	0.76 (0.42, 1.38)		0.72 (0.32, 1.66)		0.8 (0.53, 1.21)		1.19 (0.81, 1.75)		0.73 (0.51, 1.05)	
**Chronic health condition**										
No										
Yes	2.76 (1.50, 5.08)	1.86 (1.01, 3.41)	4.4 (2.86, 6.78)	4.18 (2.67, 6.54)	6.62 (4.38, 10.0)	4.71 (2.92, 7.59)	2.49 (1.61, 3.86)	2.73 (1.61, 4.62)	3.83 (2.14, 6.88)	3.16 (1.82, 5.51)

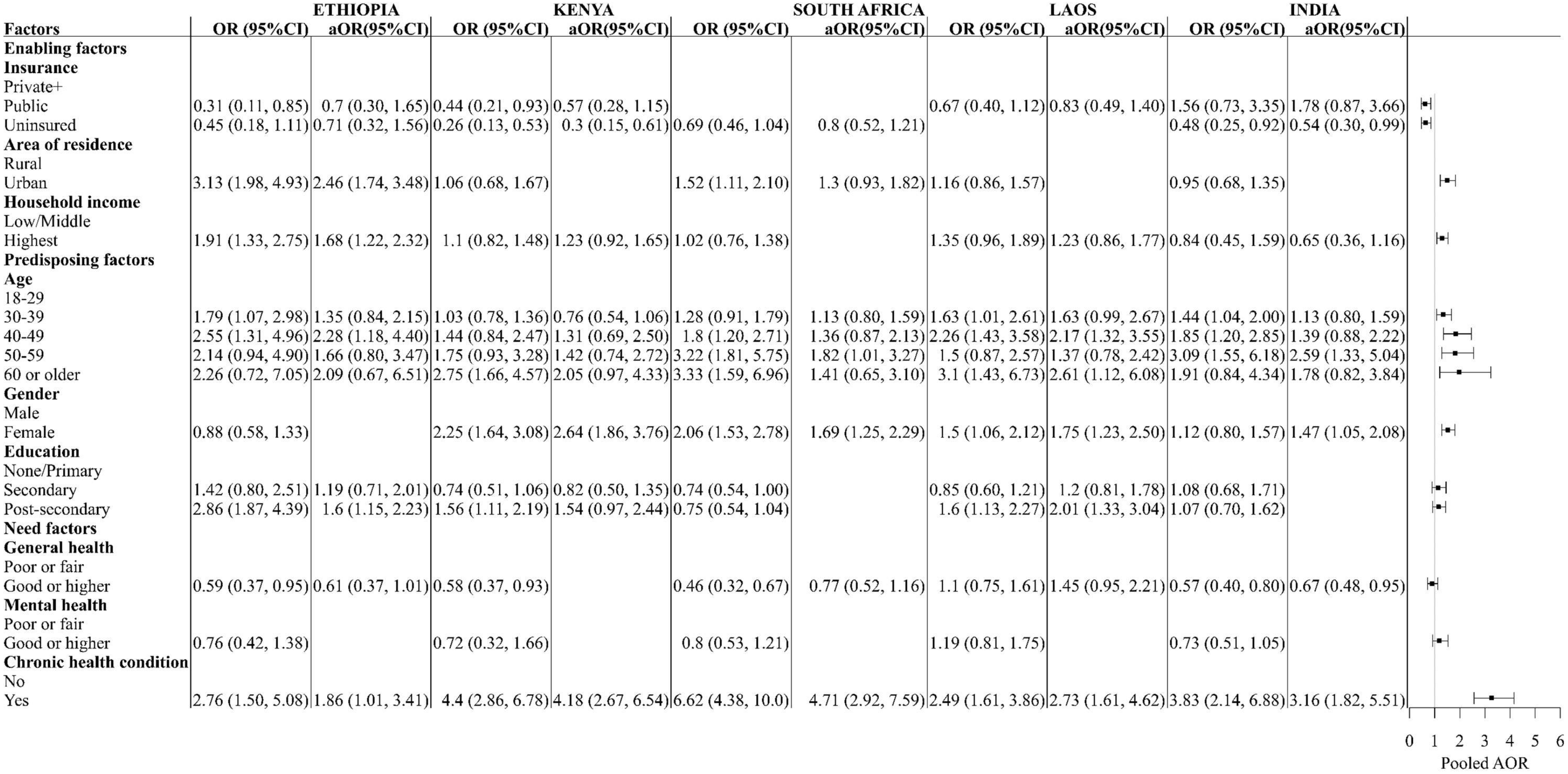

Appendix 2 figure 9: Estimated associations between type of health insurance and eye or teeth examination by countries: Odds ratio (OR) and adjusted odds ratio (aOR)

	ETHIOPIA		KENYA		SOUTH AFRICA		LAOS		INDIA	
**Factors**	**OR (95%CI)**	**aOR(95%CI)**	**OR (95%CI)**	**aOR(95%CI)**	**OR (95%CI)**	**aOR(95%CI)**	**OR (95%CI)**	**aOR(95%CI)**	**OR (95%CI)**	**aOR(95%CI)**
**Enabling factors**										
**Insurance**										
Private+										
Public	0.35 (0.16, 0.75)	0.41 (0.19, 0.87)	0.6 (0.32, 1.15)	0.57 (0.29, 1.11)			0.5 (0.33, 0.75)	0.65 (0.43, 0.98)	1.58 (0.71, 3.54)	2.17 (0.82, 5.74)
Uninsured	0.59 (0.28, 1.25)	0.66 (0.33, 1.32)	0.26 (0.14, 0.48)	0.28 (0.14, 0.55)	0.34 (0.24, 0.50)	0.41 (0.27, 0.62)			0.67 (0.34, 1.31)	0.84 (0.35, 2.05)
**Area of residence**										
Rural										
Urban	1.66 (1.11, 2.46)	1.33 (0.81, 2.17)	1.73 (0.95, 3.13)	1.58 (0.99, 2.53)	1.16 (0.84, 1.62)		1.99 (1.43, 2.75)	1.49 (1.07, 2.08)	1.38 (0.94, 2.03)	1.54 (1.05, 2.28)
**Household income**										
Low/Middle										
Highest	1.21 (0.86, 1.71)		1.29 (0.86, 1.94)		1.62 (1.20, 2.18)	1.21 (0.87, 1.69)	2.19 (1.50, 3.19)	1.82 (1.23, 2.69)	1.11 (0.55, 2.22)	
**Predisposing factors**										
**Age**										
18-29										
30-39	2.39 (1.15, 4.98)	2.1 (1.03, 4.28)	0.81 (0.53, 1.24)	0.66 (0.42, 1.06)	0.82 (0.58, 1.14)	0.83 (0.59, 1.16)	1.57 (0.93, 2.67)	1.45 (0.81, 2.58)	1.19 (0.81, 1.74)	0.94 (0.62, 1.42)
40-49	2.38 (1.20, 4.73)	2.17 (1.04, 4.49)	1.02 (0.61, 1.70)	0.86 (0.53, 1.40)	0.84 (0.56, 1.25)	0.84 (0.56, 1.27)	1.68 (1.04, 2.74)	1.56 (0.90, 2.70)	1.61 (1.00, 2.57)	1.31 (0.80, 2.16)
50-59	2.15 (0.81, 5.67)	1.89 (0.70, 5.09)	1.44 (0.58, 3.61)	1.21 (0.41, 3.51)	1.08 (0.67, 1.72)	1.09 (0.67, 1.76)	1.85 (1.07, 3.20)	1.74 (0.92, 3.27)	2.84 (1.37, 5.87)	2.28 (1.14, 4.57)
60 or older	2.84 (0.74, 10.8)	2.41 (0.68, 8.57)	1.97 (0.50, 7.77)	1.61 (0.39, 6.63)	1.69 (0.93, 3.08)	1.59 (0.84, 2.98)	1.79 (0.95, 3.37)	1.51 (0.71, 3.23)	1.48 (0.62, 3.57)	1.3 (0.56, 3.01)
**Gender**										
Male										
Female	0.92 (0.61, 1.38)		0.77 (0.52, 1.15)	0.86 (0.58, 1.28)	0.82 (0.61, 1.10)	0.78 (0.58, 1.04)	1.14 (0.80, 1.61)	1.35 (0.91, 2.01)	1.16 (0.80, 1.70)	1.39 (0.95, 2.04)
**Education**										
None/Primary										
Secondary	1.22 (0.69, 2.14)		1.1 (0.72, 1.68)		1.13 (0.83, 1.53)		1.04 (0.71, 1.52)	1.04 (0.67, 1.63)	0.89 (0.54, 1.46)	0.83 (0.50, 1.38)
Post-secondary	1.49 (0.98, 2.27)		1.67 (1.13, 2.47)		1.41 (1.02, 1.95)		2.15 (1.48, 3.12)	1.6 (0.99, 2.59)	0.84 (0.53, 1.33)	0.73 (0.46, 1.16)
**Need factors**										
**General health**										
Poor or fair										
Good or higher	0.54 (0.33, 0.90)	0.62 (0.39, 0.98)	0.8 (0.46, 1.39)	0.83 (0.58, 1.19)	0.99 (0.69, 1.42)		1.4 (0.95, 2.08)	1.53 (0.98, 2.38)	0.89 (0.60, 1.32)	
**Mental health**										
Poor or fair										
Good or higher	0.65 (0.36, 1.19)		1.03 (0.42, 2.50)		1.19 (0.78, 1.84)		1.27 (0.84, 1.92)		0.76 (0.51, 1.14)	
**Chronic health condition**										
No										
Yes	2.05 (1.25, 3.36)	1.3 (0.67, 2.52)	1.49 (0.87, 2.55)	1.47 (0.77, 2.81)	1.16 (0.81, 1.67)		1.27 (0.84, 1.92)	1.39 (0.83, 2.31)	2.46 (1.35, 4.47)	2.38 (1.33, 4.25)

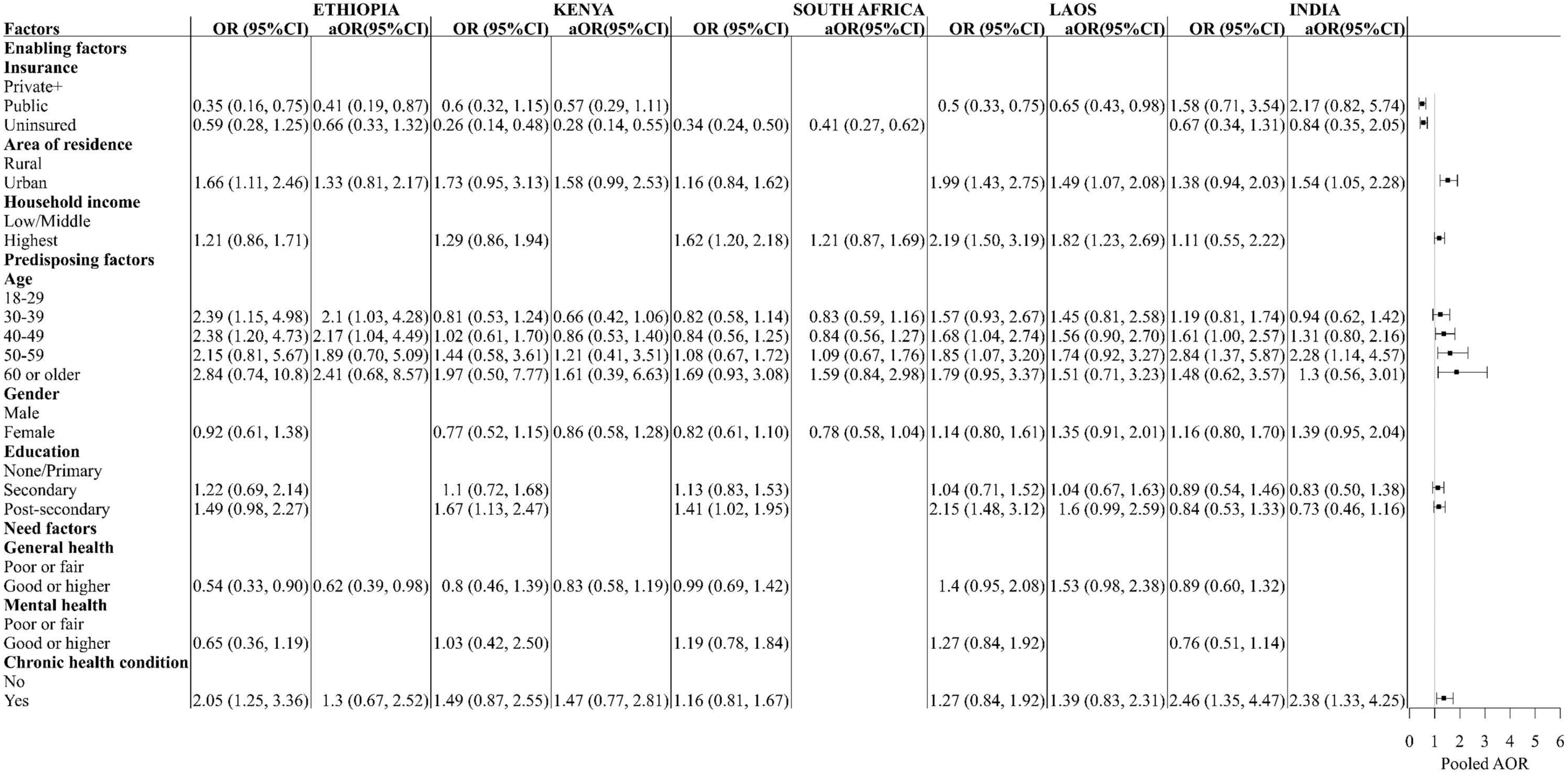

Appendix 2 figure 10: Estimated associations between type of health insurance and confidence in ability to receive and afford care by countries: Odds ratio (OR) and adjusted odds ratio (aOR)

	ETHIOPIA		KENYA		SOUTH AFRICA		LAOS		INDIA	
**Factors**	**OR (95%CI)**	**aOR(95%CI)**	**OR (95%CI)**	**aOR(95%CI)**	**OR (95%CI)**	**aOR(95%CI)**	**OR (95%CI)**	**aOR(95%CI)**	**OR (95%CI)**	**aOR(95%CI)**
**Enabling factors**										
**Insurance**										
Private+										
Public	1.67 (0.72, 3.87)	1.49 (0.70, 3.17)	0.77 (0.44, 1.37)	0.82 (0.45, 1.49)			0.43 (0.22, 0.83)	0.5 (0.26, 0.96)	1.85 (0.79, 4.36)	1.86 (0.84, 4.15)
Uninsured	1.77 (0.87, 3.64)	1.66 (0.81, 3.40)	0.59 (0.34, 1.04)	0.66 (0.36, 1.22)	0.63 (0.44, 0.90)	0.52 (0.35, 0.77)			1.44 (0.70, 2.92)	1.39 (0.72, 2.66)
**Area of residence**										
Rural										
Urban	0.87 (0.55, 1.38)	0.84 (0.58, 1.24)	1.17 (0.84, 1.62)		0.84 (0.62, 1.15)	0.85 (0.62, 1.16)	0.86 (0.62, 1.19)	0.6 (0.41, 0.87)	1.02 (0.72, 1.45)	
**Household income**										
Low/Middle										
Highest	1.19 (0.57, 2.46)	1.26 (0.61, 2.62)	1.39 (0.93, 2.07)	1.28 (0.91, 1.80)	0.99 (0.74, 1.33)		1.81 (1.27, 2.57)	1.73 (1.20, 2.49)	1.65 (0.94, 2.90)	1.76 (0.95, 3.26)
**Predisposing factors**										
**Age**										
18-29										
30-39	0.93 (0.52, 1.66)	0.93 (0.54, 1.62)	0.92 (0.64, 1.31)	0.89 (0.63, 1.26)	1.02 (0.74, 1.41)	1 (0.72, 1.38)	2.19 (1.36, 3.55)	2.13 (1.25, 3.64)	0.9 (0.64, 1.26)	0.92 (0.64, 1.32)
40-49	1.04 (0.55, 1.97)	1.1 (0.56, 2.18)	1.24 (0.75, 2.03)	1.28 (0.82, 2.01)	1.04 (0.70, 1.56)	1.04 (0.69, 1.57)	2.67 (1.65, 4.34)	2.9 (1.67, 5.02)	0.75 (0.48, 1.18)	0.82 (0.51, 1.29)
50-59	1.54 (0.67, 3.54)	1.59 (0.64, 3.97)	1.12 (0.64, 1.95)	1.21 (0.73, 2.01)	0.83 (0.52, 1.31)	0.86 (0.53, 1.39)	2.14 (1.20, 3.83)	2.16 (1.14, 4.09)	0.9 (0.42, 1.93)	0.95 (0.48, 1.85)
60 or older	0.81 (0.27, 2.47)	0.79 (0.27, 2.25)	0.87 (0.45, 1.66)	0.98 (0.56, 1.70)	1.1 (0.60, 1.99)	1.18 (0.62, 2.25)	4.9 (2.32, 10.3)	5.23 (2.17, 12.6)	1.1 (0.47, 2.59)	1.1 (0.50, 2.44)
**Gender**										
Male										
Female	0.98 (0.52, 1.83)		1.01 (0.64, 1.59)	1.14 (0.77, 1.69)	0.81 (0.61, 1.08)	0.85 (0.64, 1.13)	0.99 (0.69, 1.43)	1.19 (0.82, 1.71)	1.13 (0.80, 1.59)	
**Education**										
None/Primary										
Secondary	1.16 (0.72, 1.88)		1.28 (0.92, 1.78)		0.75 (0.56, 1.01)	0.65 (0.48, 0.88)	0.97 (0.67, 1.41)	1.42 (0.90, 2.23)	1.29 (0.80, 2.08)	1.4 (0.89, 2.20)
Post-secondary	0.92 (0.63, 1.34)		1.24 (0.86, 1.80)		0.8 (0.58, 1.10)	0.62 (0.45, 0.87)	1.13 (0.78, 1.65)	1.38 (0.88, 2.18)	1.12 (0.73, 1.72)	1.12 (0.75, 1.66)
**Need factors**										
**General health**										
Poor or fair										
Good or higher	0.77 (0.47, 1.27)	0.79 (0.50, 1.24)	1.39 (1.00, 1.93)		1.01 (0.71, 1.43)		1.86 (1.26, 2.74)	1.66 (1.01, 2.72)	1.43 (1.00, 2.05)	1.57 (1.05, 2.35)
**Mental health**										
Poor or fair										
Good or higher	0.74 (0.39, 1.42)	0.78 (0.41, 1.46)	1.83 (0.88, 3.82)	1.86 (0.93, 3.72)	1.23 (0.81, 1.88)	1.21 (0.79, 1.85)	1.92 (1.29, 2.86)	1.39 (0.85, 2.27)	0.98 (0.67, 1.43)	0.73 (0.48, 1.10)
**Chronic health condition**										
No										
Yes	0.85 (0.47, 1.52)	0.74 (0.45, 1.21)	0.73 (0.40, 1.34)	0.78 (0.44, 1.36)	0.73 (0.51, 1.05)	0.71 (0.49, 1.03)	1.19 (0.78, 1.80)	1.25 (0.77, 2.03)	0.69 (0.38, 1.27)	0.75 (0.41, 1.38)

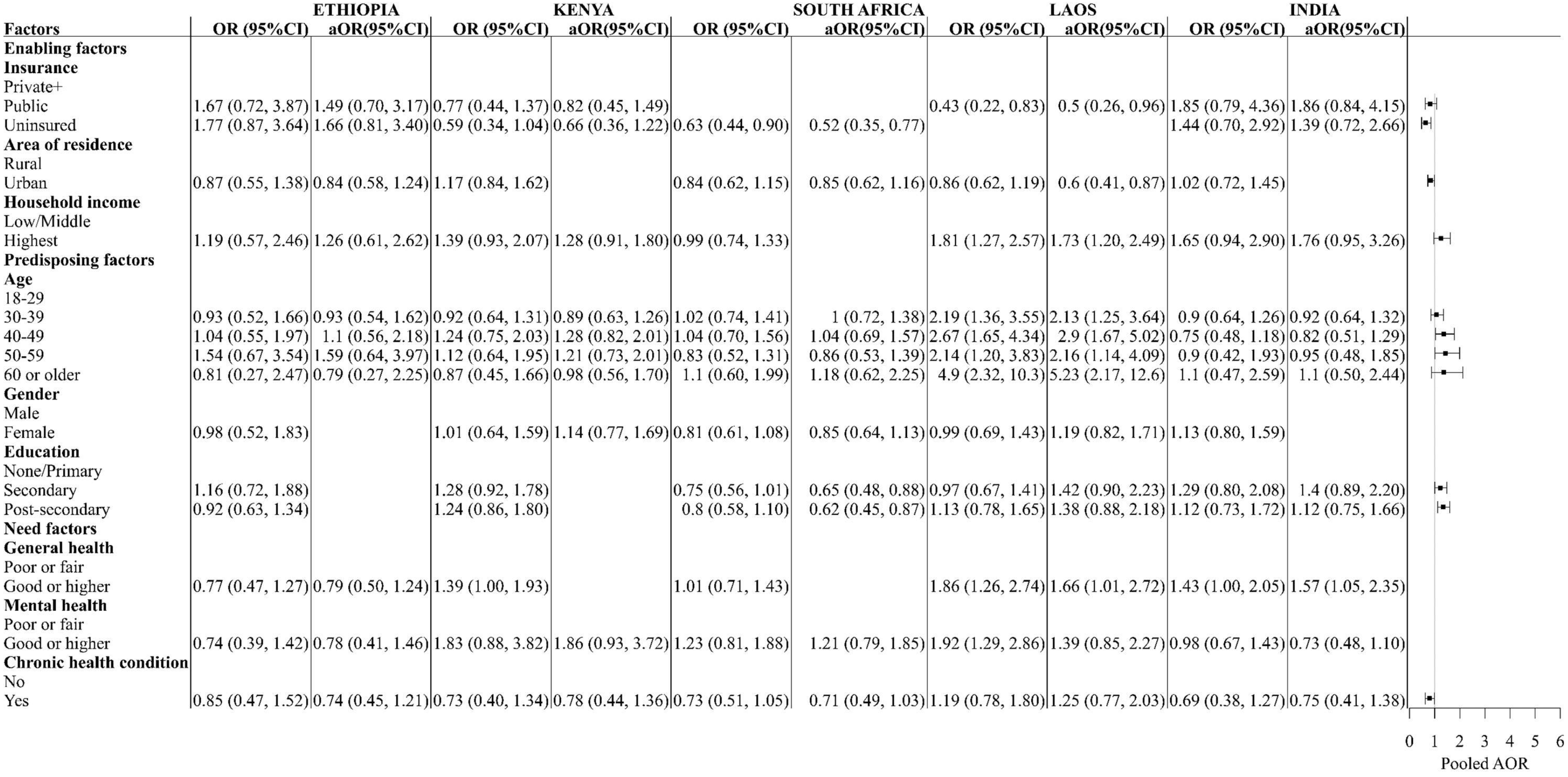

Supplement: Supplementary appendix 2 [file mmc2.pdf]
